# A novel Betacoronavirus characterised in collared peccaries from the Rio de Janeiro Zoo (Brazil) killed by unknown disease

**DOI:** 10.1590/0074-02760200153

**Published:** 2020-08-10

**Authors:** Mirela D’arc, Matheus Calvano Cosentino, Filipe Romero Rebello Moreira, Liliane Tavares Faria Cavalcante, Anderson Mendes Augusto, Fernando Trocolli, Daniel Guimarães Ubiali, Carlos Eduardo Verona, Marcelo Alves Soares, André Felipe Santos

**Affiliations:** 1 Universidade Federal do Rio de Janeiro, Rio de Janeiro, RJ, Brasil; 2 Zoológico do Rio de Janeiro S/A, Rio de Janeiro, RJ, Brasil; 3 Universidade Federal Rural do Rio de Janeiro, Seropédica, RJ, Brasil; 4 Instituto Nacional de Câncer, Rio de Janeiro, RJ, Brasil

**Keywords:** virome, Pecari tajacu, Betacoronavirus, zoonoses, one health

## Abstract

In an enclosure with nine collared peccaries (*Pecari tajacu*) from the Rio de Janeiro city Zoo, Brazil, one specimen was found dead and two others developed prostration, apathy and dehydration, resulting on its death. Necropsy of two animals pointed to pulmonary and renal damage. Histological examination revealed vasculitis in spleen from both *P. tajacu*, suggesting a systemic viral infection. Lungs from one specimen showed fibrinoid vasculitis, alveolar damage with hyaline membrane, and interstitial lymphocytes infiltration. Virome analysis in anal wash samples from the latter two animals revealed a new type of Betacoronavirus, lineage A, provisionally named Ptajacu-CoV.

On September 12, 2018, one collared peccary (*Pecari tajacu*), number 6640, elderly male, residing in Zoológico do Rio de Janeiro (RioZoo), was hospitalised due to an intense prostration, apathy and dehydration verified by physical examination of the mucous membranes and capillary fill time. Two days later, a second animal (#451), elderly female, of the same enclosure was found dead; a third peccary (#8515), elderly male, was hospitalised with the same clinical signs of specimen #6640 in addition to haematochezia. Specimens #6640 and #8515 died after eight and six days of fluid therapy, analgesia, and antibiotic therapy, respectively. The other six peccaries that lived in the same enclosure that had contact with the sick animals were all adults and showed no clinical signs. The haemogram of both specimens #6640 and #8515 showed moderate neutrophil left-shift, indicating inflammation. A high rate of potassium was also found, indicating a possible renal malfunction. The blood culture did not show evidence of bacterial infection. The necropsy reports of the three animals showed poor score condition, excessive tooth wear, kidneys with multifocal white spots and a severe foamy oedema in the trachea and lungs.

A set of tissues (brain, kidney, liver, spleen, trachea, lung, heart, and intestines) was collected from animals #451 and #6640 and fixed in 10% formalin for histopathology. Tissues were stained with haematoxylin and eosin, and the kidney from animal #451 was stained with Congo red and Wharthin-Starry. Histological examination revealed, in both specimens, spleens with multiple vessels with eosinophilic amorphous wall and pyknotic nuclei (fibrinoid vasculitis) and mild lymphoid necrosis. There were also lung oedema and hepatocellular fatty degeneration. The lung from #6640 showed multifocal moderate alveolar damage with hyaline membrane formation. Interstitial infiltrate dominated by lymphocytes was also seen. The kidneys from #6640 had pyelonephritis with interstitial and tubular infiltrate, mainly in the medulla, with neutrophils, lymphocytes, histiocytes, and plasma cells. In the cortical kidney, the glomeruli were surrounded by a lamellated, thickened, Bowman capsular basement membrane and layers of collagen (periglomerular fibrosis). The kidneys from #451 revealed mild lymphoplasmacytic nephritis, periglomerular fibrosis, and the glomeruli had pink amorphous material deposition in extracellular space. Congo red stain confirmed the presence of amyloid protein in these glomeruli. Wharthin-Starry stain failed to reveal any spirochete bacteria in the kidneys.

Blood cultures failed to reveal any systemic bacterial infection, as well as the Wharthin-Starry stain allowed to rule out the suspicion of leptospirosis. Thus, we sought to a possible viral infection through massive sequencing. Samples of anal wash were collected from the two hospitalised peccaries using 5 mL of sterile phosphate buffer injected into the animals’ anal orifice based on federal technical registration 6690176 for handling wild animals. Samples were stored in 15 mL sterile tubes with 5 mL of RNAlater® solution. Preparation steps included sample mixing (500 µL of each), filtering, ultracentrifugation and enzymatic digestion. Total nucleic acids were extracted and cDNA amplification was prepared for library construction. Massive sequencing was conducted in a MiSeq Illumina® platform. The pipeline used FASTQC and Sickle, for quality and size control; SAMTOOLS and BWA, with the reference genome of *Sus scrofa domesticus* (GCF_000003025.6), to remove putative host’s reads; and, two rounds of BLASTp analysis, one against an in-house viral database and another against total non-redundant GenBank^®^ database only for the annotated reads. Geneious Prime^®^ v.2019.2.3 was used to generate viral consensus sequences using reference-based assembly. A total of 10,693,960 reads were obtained and 1,967 were assigned to three virus families: *Myoviridae* (15 reads), *Papillomaviridae* (25 reads) and *Coronaviridae* (1,927 reads). Using a porcine coronavirus reference genome, PHEV (DQ011855), a consensus sequence of 30,492 bp was assembled and shown to be a novel strain with 94% of similarity to the reference (Supplementary data). Mapped reads showed 50-251pb with coverage of 9.52x and standard deviation of 7.19 [Supplementary data **(Figure)**]. Primers were designed and used to confirm the presence of virus in both peccary samples by nested polymerase chain reaction (PCR) and Sanger sequencing. CoV-positive samples were identified by PCR, adding 2.5 μL of cDNA to a mix containing 1x High Fidelity Buffer, 2 mM MgSO_4_, 0.2 pmol of each primer, 0.2 mM dNTP mix and 0.5 U *Platinum™ Taq DNA Polymerase High Fidelity* (Thermo-Fisher Scientific), totalling 25 μL. Reactions were carried out with an initial denaturation at 95ºC for 10 min, followed by 35 cycles of denaturation at 95ºC for 45 sec, annealing at specific primer temperatures [Supplementary data **(Table)**] for 1 min and extension at 72ºC for 3 min, with a final extension at 72ºC for 10 min.

A 1,280 bp fragment of viral RNA polymerase amplified in both animals showed a 100% identity, suggesting that the same virus strain circulated in both peccaries, provisionally named Ptajacu-CoV. From the consensus, a genomic fragment equivalent to viral polymerase (17,592 bp) was used in alignment with other sequences of coronavirus and the phylogenetic analysis grouped this novel strain within lineage A of *Betacoronavirus* genus (Figure), including the human viral strains OC43 and HKU1, and several ungulate mammal viruses.

**Figure:**
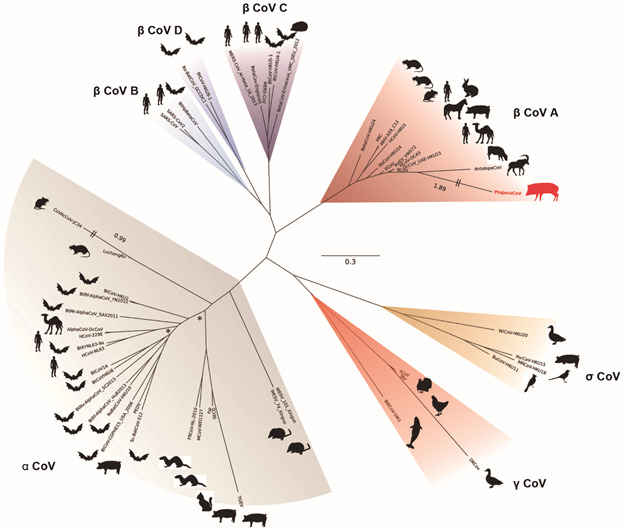
Maximum likelihood phylogeny inferred under the GTR+F+I+G4 model from an open reading frame (ORF) 1AB alignment (17,592 bp, after trimming of sites with more than 10% of gaps), comprehending coronavirus sequences available on NCBI RefSeq and the novel Ptajacu-CoV. Different genera or Betacoronavirus clades are colour labelled and asterisks mark nodes inferred with low support values (SH-aLRT < 0.70). Branch lengths bigger than 0.7 have been trimmed and annotated to keep figure dimensions. Alphacoronavirus (a CoV): BtRf-AlphaCoV-YN2012 (NC_028824, bat), BtCoV-HKU2 (NC_009988, bat), AlphaCoV-DcCoV (NC_028752, camel), HCoV-229E (NC_002645, human), BtCoV-HKU8 (NC_010438, bat), BtCoV-1A (NC_010437, bat), BtNv-AlphaCoV-SC2013 (NC_028833, bat), HCoV-NL63 (NC_005831, human), BtRf-AlphaCoV-HuB2013 (NC_028814, bat), Sc-BatCoV-512 (NC_009657, bat), PEDV (NC_003436, pig), BtCoV-CDPHE15 (NC_022103, bat), LuchengRn (NC_032730, rat), MCoV-WD1127 (NC_023760, mink), SECD (NC_028806, pig) and FIP (NC_002306, cat). Gammacoronavirus (γ CoV): BWCoV-SW1 (NC_010646, beluga whale), IBV (NC_001451, chicken), TCoV (NC_010800, turkey). Deltacoronavirus (δ CoV): WiCoV-HKU20 (JQ065048, duck), BuCoV-HKU11 (FJ376619, bulbul), PorCoV-HKU15 (JQ065042, pig), MRCoV-HKU18 (JQ065046, magpie-robin). Betacoronavirus lineage A (ꞵ CoV A): HCoV-HKU1 (NC_006577, human), PRC (NC_012936, rat), MHV-A59-C12 (NC_001846, mouse), BetaCoV-HKU24 (NC_026011, rat), RbCoV-HKU14 (NC_017083, rabbit), HCoV-OC43 (NC_006213, human), PHEV-VW572 (DQ011855, pig), AntelopeCoV (EF424621, sable antelope), BCoV (NC_003045, cow), Ptajacu-CoV (MT083879, collared peccary), ECoV (NC_010327.1, horse), DcCoV (KF906251, camel). Betacoronavirus lineage B (ꞵ CoV B): 2019_nCOV (NC_045512.2, human), SARS-CoV (NC_004718, human). Betacoronavirus lineage C (ꞵ CoV C): BetaCoV-Erinaceus (NC_039207, hedgehog), BtCoV-HKU4-1 (NC_009019, bat), BtCoV-HKU5-1 (NC_009020, bat), MERS-CoV (NC_019843, human), BetaCov-England-1 (NC_038294, human). Betacoronavirus lineage D (ꞵ CoV D): BtCoV-HKU9-1 (NC_009021, bat), Ro-BatCoV (NC_030886, bat).

*Coronaviridae* family is composed of enveloped viruses with the largest known RNA genome (26-32 Kb) and it is divided in four genera infecting a wide-range of mammals and birds.^1^ Betacoronaviruses have a great importance in global human health, being responsible from the common cold and pneumonia to severe respiratory diseases, this last caused by emerging viruses severe acute respiratory syndrome coronavirus (SARS-CoV) (lineage B), Middle East respiratory syndrome Corona virus (MERS-CoV) (lineage C) and the recent SARS-CoV-2 (lineage B).^2^ Despite lineage A is related in humans only with common cold and pneumonia, ungulate animals have already been described with neonatal diarrhoea associated with BCoV (infecting bovines), DcCoV (dromedaries), ECoV (equines), and PHEV (pigs) infections.^3^ ^,^ ^4^ ^,^ ^5^ PHEV can also cause vomiting, constipation, wasting, respiratory signs, decreased weight gain and neurologic signs including ataxia, stiffness, hyperesthesia, and posterior paralysis and death in piglets less than 4-weeks-old.^6^ Herein, peccaries presented prostration, apathy and damages in lungs, liver and kidneys. The vasculitis observed in the spleen of two specimens suggested a systemic viral infection. In addition, pulmonary damage in one specimen resembles the one seen upon SARS-CoV infection of humans. However, the observed kidney lesions differed from other coronavirus infections.^7^ This report is the first case of a wild pig infection with coronavirus from American continent. Ungulates infected with lineage A betacoronavirus are generally cubs. Possibly, adult peccaries are capable of dealing with the viral infection, while elderly animals, with their natural immunosenescence, developed the most severe condition in response to viral replication.^8^ This is the first report of elderly animals infected with a coronavirus that may have led to a deadly disease, strengthening that wild animal centres and zoos should adopt additional care in the management of elderly animals.

Zoonotic transmission is a common characteristic of coronavirus, as proven recently by MERS-CoV, SARS-CoV and SARS-CoV-2 from bat coronaviruses.^9^ PHEV, BCoV and human OC43 had the most recent common ancestor dated in the early 1900s from an unknown origin.^10^ Sequencing the complete Ptajacu-CoV genome will help to elucidate its origin. In the context of an One Health approach, the characterisation of novel viruses, especially coronaviruses in animals might contribute to guide new policies for zoonotic disease control, in addition to protecting endangered animals.^11^ ^,^ ^12^
