# Supplementary material for: A novel Betacoronavirus characterised in collared peccaries from the Rio de Janeiro Zoo (Brazil) killed by unknown disease
Source: Mem Inst Oswaldo Cruz. 2020 Aug 10;115:e200153. doi: 10.1590/0074-02760200153 (PMC7416640; doi:10.1590/0074-02760200153)
Supplement: Supplementary file 1 [file 1678-8060-mioc-115-e200153-s.pdf]

TABLE

Primer list used for CoV nested-PCR and Sanger sequencing. The detection of CoV in pooled library samples and the complete open reading frame (ORF) 1a&b region were amplified from individual samples with those specifically designed primers. Primers were designed with *Primer3* in the Geneious Prime 2019.2.3. sotware, used to confirm the presence of virus in both peccary samples by nested PCR and Sanger sequencing and to complete regions with no coverage

Primer name	Sequence 5' - 3' (bp)	Annealing temperature (°C)	Fragment (bp)
Ptajacu-CoV - 787 F<br>Ptajacu-CoV - 2,769 R	GCGTAAGTGTGGCGAAAAGG<br>CCTTCCAGCACAAGGAACCC	52	1982
Ptajacu-CoV - 3,175 F<br>Ptajacu-CoV - 5,310 R	GTCACAAGTGCTGGAGAGCC<br>ACGAAATTCAAGCCACGCCT	53	2135
Ptajacu-CoV - 5,339 F<br>Ptajacu-CoV - 7,712 R	TGGTTTTGGCCAAAGGTGGG<br>TATGTGCGCTGTCCATCACG	57	2373
Ptajacu-CoV - 6,890 F<br>Ptajacu-CoV - 8,170 R	TCGGTTTCTTGCCGACTTTTGT<br>CCGTCAATTCAAGGCCTGCA	57	1280
Ptajacu-CoV - 9,209 F<br>Ptajacu-CoV - 11,693 R	TGCCTGGGACCTTTTGTGG<br>GGCACACCTCCAATACCCA	57	2484
Ptajacu-CoV - 12,058 F<br>Ptajacu-CoV - 14,293 R	GCTCGTTCTAGTGGTTCTGC<br>CTAACAAGAGGCCCAAAACATG	55	2865
Ptajacu-CoV - 13,903 F<br>Ptajacu-CoV - 16,131 R	GGTGGAGGTAGGCTTGGTAG<br>GAGAAGAGCAGACCACGCAA	61	2228
Ptajacu-CoV - 16,733 F<br>Ptajacu-CoV - 18,590 R	CGCGCCACAACCACTTATAAG<br>GCGCCAACAACCATAGTAACC	61	1857
Ptajacu-CoV - 18,835 F<br>Ptajacu-CoV - 21,088 R	GGGTCTTGCAGCGTGTTATG<br>CCCAGAGCTAACTTGTCGC	60	2253
Ptajacu-CoV - 20,103 F<br>Ptajacu-CoV - 22,628 R	GTCGGTGGTAATGATGCTC<br>CAAAGGGCTGTAGGTTGTGC	55	2525

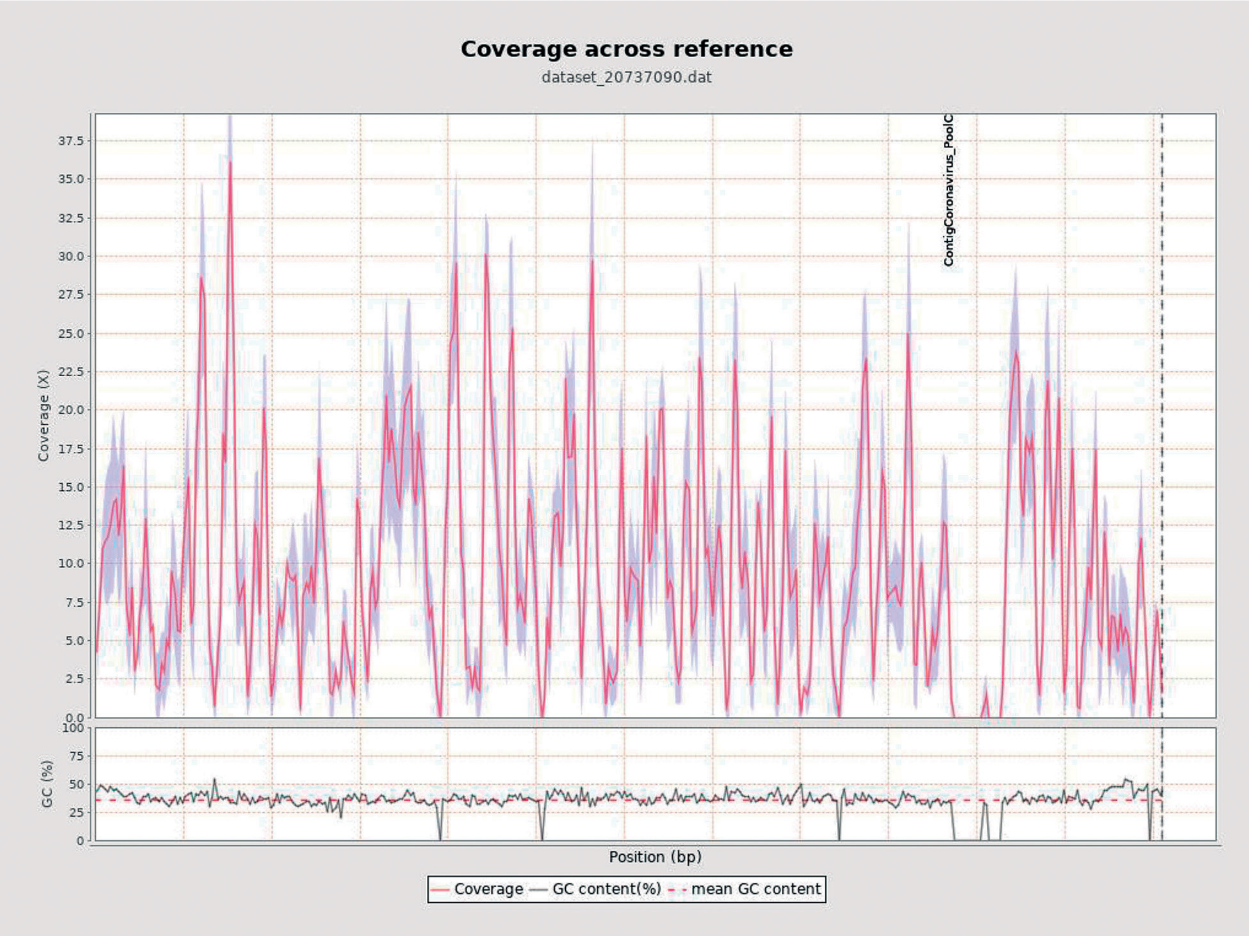

Coverage of Ptajacu-CoV reads obtained using the standard parameters of the QualiMap BamQC tool (Galaxy Version 2.2.2c+galaxy1) available in the Galaxy web platform.

ORF,Gene,p-distances,% Reference covered
orf1,Replicase,0.0466847090663058,0.972003006388576
orf2,NS2,0.0735042735042735,1
orf3,HE,0.101255886970173,0.99921568627451
orf4,S,0.121212121212121,0.66
orf5,NS9.4,0.15625,0.853333333333333
orf6,NS12.7,0.115853658536585,0.993939393939394
orf7,E,0.0274509803921569,1
orf8,M,0.0836940836940837,1
orf9,N,0.0515695067264574,0.991111111111111
orf10,N2,0.0453074433656958,0.990384615384615

ORF,Protein,ORF_POSITION (PHEV),NT_DIFFERENCE(PHEV->Ptjacu-CoV),TYPE,AA_DIFFERENCE(PHEV->Ptjacu-CoV)
orf1.fasta,Replicase protein,34, t -> c, N, Y -> H
orf1.fasta,Replicase protein,87, t -> c, S, NA
orf1.fasta,Replicase protein,117, t -> a, S, NA
orf1.fasta,Replicase protein,207, g -> a, S, NA
orf1.fasta,Replicase protein,261, c -> t, S, NA
orf1.fasta,Replicase protein,300, t -> c, S, NA
orf1.fasta,Replicase protein,372, c -> t, S, NA
orf1.fasta,Replicase protein,474, t -> a, S, NA
orf1.fasta,Replicase protein,486, c -> t, S, NA
orf1.fasta,Replicase protein,501, c -> t, S, NA
orf1.fasta,Replicase protein,536, c -> t, N, A -> V
orf1.fasta,Replicase protein,567, c -> t, S, NA
orf1.fasta,Replicase protein,591, t -> a, N, D -> E
orf1.fasta,Replicase protein,606, t -> c, S, NA
orf1.fasta,Replicase protein,670, c -> t, N, Q -> L
orf1.fasta,Replicase protein,671, a -> t, N, Q -> L
orf1.fasta,Replicase protein,704, g -> a, N, R -> K
orf1.fasta,Replicase protein,719, t -> k, N, L -> X
orf1.fasta,Replicase protein,720, a -> m, N, L -> X
orf1.fasta,Replicase protein,721, c -> m, N, L -> X
orf1.fasta,Replicase protein,722, t -> w, N, L -> X
orf1.fasta,Replicase protein,723, t -> y, N, L -> X
orf1.fasta,Replicase protein,724, a -> m, N, R -> X
orf1.fasta,Replicase protein,727, g -> k, N, G -> X
orf1.fasta,Replicase protein,753, c -> t, S, NA
orf1.fasta,Replicase protein,861, c -> t, S, NA
orf1.fasta,Replicase protein,884, c -> t, N, T -> I
orf1.fasta,Replicase protein,909, t -> w, N, D -> X
orf1.fasta,Replicase protein,912, a -> r, N, P -> X
orf1.fasta,Replicase protein,928, t -> c, S, NA
orf1.fasta,Replicase protein,933, g -> a, S, NA
orf1.fasta,Replicase protein,958, g -> a, N, A -> T
orf1.fasta,Replicase protein,1011, a -> g, S, NA
orf1.fasta,Replicase protein,1081, t -> k, N, C -> X
orf1.fasta,Replicase protein,1173, t -> c, S, NA
orf1.fasta,Replicase protein,1215, g -> t, S, NA
orf1.fasta,Replicase protein,1242, c -> t, S, NA
orf1.fasta,Replicase protein,1263, a -> g, S, NA
orf1.fasta,Replicase protein,1320, c -> t, S, NA
orf1.fasta,Replicase protein,1338, t -> c, S, NA
orf1.fasta,Replicase protein,1371, t -> c, S, NA
orf1.fasta,Replicase protein,1372, c -> a, N, L -> M
orf1.fasta,Replicase protein,1395, c -> t, S, NA
orf1.fasta,Replicase protein,1455, c -> t, S, NA
orf1.fasta,Replicase protein,1474, a -> g, N, S -> G
orf1.fasta,Replicase protein,1508, c -> m, N, A -> X
orf1.fasta,Replicase protein,1511, t -> k, N, I -> X
orf1.fasta,Replicase protein,1516, a -> r, N, K -> X
orf1.fasta,Replicase protein,1518, g -> k, N, K -> X

orf1.fasta,Replicase protein,1519, g -> s, N, E -> X
orf1.fasta,Replicase protein,1520, a -> w, N, E -> X
orf1.fasta,Replicase protein,1521, a -> m, N, E -> X
orf1.fasta,Replicase protein,1522, a -> r, N, T -> X
orf1.fasta,Replicase protein,1523, c -> s, N, T -> X
orf1.fasta,Replicase protein,1524, a -> w, N, T -> X
orf1.fasta,Replicase protein,1525, a -> r, N, N -> X
orf1.fasta,Replicase protein,1561, g -> c, N, E -> Q
orf1.fasta,Replicase protein,1590, a -> g, S, NA
orf1.fasta,Replicase protein,1734, c -> t, S, NA
orf1.fasta,Replicase protein,1761, a -> g, S, NA
orf1.fasta,Replicase protein,1797, t -> a, N, H -> Q
orf1.fasta,Replicase protein,1809, t -> c, S, NA
orf1.fasta,Replicase protein,1812, t -> c, S, NA
orf1.fasta,Replicase protein,1822, c -> a, N, L -> I
orf1.fasta,Replicase protein,1824, t -> c, N, L -> I
orf1.fasta,Replicase protein,1833, c -> t, S, NA
orf1.fasta,Replicase protein,1841, c -> t, N, S -> F
orf1.fasta,Replicase protein,1854, c -> g, N, D -> E
orf1.fasta,Replicase protein,1867, t -> g, N, S -> A
orf1.fasta,Replicase protein,1869, c -> t, N, S -> A
orf1.fasta,Replicase protein,1881, g -> a, S, NA
orf1.fasta,Replicase protein,1882, g -> a, N, G -> S
orf1.fasta,Replicase protein,1907, t -> c, N, F -> S
orf1.fasta,Replicase protein,1948, g -> a, N, A -> T
orf1.fasta,Replicase protein,1964, c -> a, N, T -> K
orf1.fasta,Replicase protein,1973, g -> a, N, R -> H
orf1.fasta,Replicase protein,1976, a -> t, N, Y -> F
orf1.fasta,Replicase protein,1977, t -> c, N, Y -> F
orf1.fasta,Replicase protein,1983, g -> a, S, NA
orf1.fasta,Replicase protein,2022, g -> t, S, NA
orf1.fasta,Replicase protein,2023, c -> t, S, NA
orf1.fasta,Replicase protein,2049, t -> c, S, NA
orf1.fasta,Replicase protein,2137, g -> t, N, V -> F
orf1.fasta,Replicase protein,2151, t -> c, S, NA
orf1.fasta,Replicase protein,2162, c -> g, N, T -> S
orf1.fasta,Replicase protein,2223, a -> t, N, R -> S
orf1.fasta,Replicase protein,2269, c -> m, N, P -> X
orf1.fasta,Replicase protein,2280, a -> t, S, NA
orf1.fasta,Replicase protein,2367, g -> a, S, NA
orf1.fasta,Replicase protein,2376, a -> g, S, NA
orf1.fasta,Replicase protein,2405, c -> m, N, P -> X
orf1.fasta,Replicase protein,2409, t -> k, N, C -> X
orf1.fasta,Replicase protein,2410, g -> r, N, G -> X
orf1.fasta,Replicase protein,2411, g -> s, N, G -> X
orf1.fasta,Replicase protein,2412, t -> m, N, G -> X
orf1.fasta,Replicase protein,2413, t -> k, N, Y -> X
orf1.fasta,Replicase protein,2422, c -> s, N, P -> X
orf1.fasta,Replicase protein,2431, t -> g, N, F -> V
orf1.fasta,Replicase protein,2439, t -> a, N, D -> E

orf1.fasta,Replicase protein,2510, a -> g, N, D -> G
orf1.fasta,Replicase protein,2517, t -> a, S, NA
orf1.fasta,Replicase protein,2541, a -> s, N, R -> X
orf1.fasta,Replicase protein,2542, g -> s, N, V -> X
orf1.fasta,Replicase protein,2547, t -> w, N, P -> X
orf1.fasta,Replicase protein,2548, t -> k, N, C -> X
orf1.fasta,Replicase protein,2549, g -> s, N, C -> X
orf1.fasta,Replicase protein,2550, t -> w, N, C -> X
orf1.fasta,Replicase protein,2551, g -> s, N, A -> X
orf1.fasta,Replicase protein,2552, c -> m, N, A -> X
orf1.fasta,Replicase protein,2553, t -> w, N, A -> X
orf1.fasta,Replicase protein,2558, a -> s, N, K -> X
orf1.fasta,Replicase protein,2559, g -> s, N, K -> X
orf1.fasta,Replicase protein,2561, g -> k, N, R -> X
orf1.fasta,Replicase protein,2562, t -> y, N, R -> X
orf1.fasta,Replicase protein,2563, g -> s, N, V -> X
orf1.fasta,Replicase protein,2564, t -> w, N, V -> X
orf1.fasta,Replicase protein,2566, a -> r, N, T -> X
orf1.fasta,Replicase protein,2568, a -> w, N, T -> X
orf1.fasta,Replicase protein,2600, t -> c, N, V -> A
orf1.fasta,Replicase protein,2606, t -> c, N, M -> T
orf1.fasta,Replicase protein,2607, g -> a, N, M -> T
orf1.fasta,Replicase protein,2670, g -> a, S, NA
orf1.fasta,Replicase protein,2688, g -> a, S, NA
orf1.fasta,Replicase protein,2715, t -> g, N, F -> L
orf1.fasta,Replicase protein,2728, a -> g, N, I -> V
orf1.fasta,Replicase protein,2805, g -> a, S, NA
orf1.fasta,Replicase protein,2814, t -> g, N, D -> E
orf1.fasta,Replicase protein,2844, c -> t, S, NA
orf1.fasta,Replicase protein,2847, t -> c, S, NA
orf1.fasta,Replicase protein,2857, t -> c, N, F -> L
orf1.fasta,Replicase protein,2862, t -> c, S, NA
orf1.fasta,Replicase protein,2898, a -> t, N, E -> D
orf1.fasta,Replicase protein,2901, c -> t, S, NA
orf1.fasta,Replicase protein,2902, t -> g, N, Y -> D
orf1.fasta,Replicase protein,2910, c -> t, S, NA
orf1.fasta,Replicase protein,2921, a -> g, N, D -> G
orf1.fasta,Replicase protein,2998, a -> g, N, K -> E
orf1.fasta,Replicase protein,3004, a -> g, N, K -> E
orf1.fasta,Replicase protein,3067, t -> w, N, S -> X
orf1.fasta,Replicase protein,3068, c -> m, N, S -> X
orf1.fasta,Replicase protein,3150, t -> g, N, D -> E
orf1.fasta,Replicase protein,3174, c -> t, S, NA
orf1.fasta,Replicase protein,3175, a -> g, N, I -> V
orf1.fasta,Replicase protein,3210, t -> y, N, T -> X
orf1.fasta,Replicase protein,3211, c -> m, N, R -> X
orf1.fasta,Replicase protein,3217, a -> w, N, N -> X
orf1.fasta,Replicase protein,3256, t -> c, S, NA
orf1.fasta,Replicase protein,3293, t -> k, N, L -> X
orf1.fasta,Replicase protein,3393, t -> c, S, NA

orf1.fasta,Replicase protein,3411, t -> c, S, NA
orf1.fasta,Replicase protein,3438, a -> t, S, NA
orf1.fasta,Replicase protein,3519, t -> c, S, NA
orf1.fasta,Replicase protein,3528, a -> g, S, NA
orf1.fasta,Replicase protein,3565, a -> g, N, N -> D
orf1.fasta,Replicase protein,3620, t -> c, N, I -> T
orf1.fasta,Replicase protein,3654, c -> t, S, NA
orf1.fasta,Replicase protein,3672, t -> c, S, NA
orf1.fasta,Replicase protein,3714, t -> c, S, NA
orf1.fasta,Replicase protein,3718, a -> g, N, I -> V
orf1.fasta,Replicase protein,3765, t -> c, S, NA
orf1.fasta,Replicase protein,3831, g -> a, S, NA
orf1.fasta,Replicase protein,3861, g -> t, S, NA
orf1.fasta,Replicase protein,3894, c -> t, S, NA
orf1.fasta,Replicase protein,3903, c -> t, S, NA
orf1.fasta,Replicase protein,3914, c -> t, N, A -> V
orf1.fasta,Replicase protein,3924, t -> a, S, NA
orf1.fasta,Replicase protein,3948, a -> g, S, NA
orf1.fasta,Replicase protein,4002, t -> a, S, NA
orf1.fasta,Replicase protein,4017, g -> a, S, NA
orf1.fasta,Replicase protein,4032, c -> t, S, NA
orf1.fasta,Replicase protein,4083, a -> t, S, NA
orf1.fasta,Replicase protein,4086, a -> g, S, NA
orf1.fasta,Replicase protein,4087, g -> a, N, A -> T
orf1.fasta,Replicase protein,4116, g -> a, S, NA
orf1.fasta,Replicase protein,4134, c -> t, S, NA
orf1.fasta,Replicase protein,4216, c -> t, S, NA
orf1.fasta,Replicase protein,4232, c -> a, N, T -> K
orf1.fasta,Replicase protein,4233, t -> g, N, T -> K
orf1.fasta,Replicase protein,4266, g -> t, N, E -> D
orf1.fasta,Replicase protein,4329, g -> a, S, NA
orf1.fasta,Replicase protein,4347, c -> a, S, NA
orf1.fasta,Replicase protein,4350, a -> g, S, NA
orf1.fasta,Replicase protein,4353, c -> t, S, NA
orf1.fasta,Replicase protein,4359, t -> c, S, NA
orf1.fasta,Replicase protein,4365, t -> c, S, NA
orf1.fasta,Replicase protein,4387, c -> a, N, L -> I
orf1.fasta,Replicase protein,4401, a -> c, N, E -> D
orf1.fasta,Replicase protein,4416, t -> g, S, NA
orf1.fasta,Replicase protein,4428, t -> c, S, NA
orf1.fasta,Replicase protein,4440, g -> t, S, NA
orf1.fasta,Replicase protein,4462, a -> g, N, I -> A
orf1.fasta,Replicase protein,4463, t -> c, N, I -> A
orf1.fasta,Replicase protein,4464, t -> a, N, I -> A
orf1.fasta,Replicase protein,4470, a -> t, N, E -> D
orf1.fasta,Replicase protein,4485, c -> t, S, NA
orf1.fasta,Replicase protein,4507, a -> g, N, I -> V
orf1.fasta,Replicase protein,4512, c -> a, S, NA
orf1.fasta,Replicase protein,4518, a -> g, S, NA
orf1.fasta,Replicase protein,4530, c -> t, S, NA

orf1.fasta,Replicase protein,4536, c -> t, S, NA
orf1.fasta,Replicase protein,4539, a -> g, S, NA
orf1.fasta,Replicase protein,4542, c -> t, S, NA
orf1.fasta,Replicase protein,4543, g -> t, N, D -> Y
orf1.fasta,Replicase protein,4572, a -> g, S, NA
orf1.fasta,Replicase protein,4581, a -> g, S, NA
orf1.fasta,Replicase protein,4605, t -> c, S, NA
orf1.fasta,Replicase protein,4613, g -> a, N, G -> D
orf1.fasta,Replicase protein,4620, t -> c, S, NA
orf1.fasta,Replicase protein,4637, g -> a, N, R -> Q
orf1.fasta,Replicase protein,4642, g -> a, N, V -> N
orf1.fasta,Replicase protein,4643, t -> a, N, V -> N
orf1.fasta,Replicase protein,4665, a -> t, S, NA
orf1.fasta,Replicase protein,4684, g -> t, N, V -> L
orf1.fasta,Replicase protein,4699, g -> a, N, V -> I
orf1.fasta,Replicase protein,4707, t -> a, S, NA
orf1.fasta,Replicase protein,4722, t -> c, S, NA
orf1.fasta,Replicase protein,4725, c -> t, S, NA
orf1.fasta,Replicase protein,4730, c -> t, N, T -> I
orf1.fasta,Replicase protein,4734, t -> c, S, NA
orf1.fasta,Replicase protein,4740, c -> t, S, NA
orf1.fasta,Replicase protein,4741, a -> g, N, I -> V
orf1.fasta,Replicase protein,4743, t -> g, N, I -> V
orf1.fasta,Replicase protein,4749, t -> a, S, NA
orf1.fasta,Replicase protein,4750, a -> g, N, S -> G
orf1.fasta,Replicase protein,4764, c -> t, S, NA
orf1.fasta,Replicase protein,4769, t -> g, N, I -> S
orf1.fasta,Replicase protein,4776, t -> a, S, NA
orf1.fasta,Replicase protein,4806, t -> g, S, NA
orf1.fasta,Replicase protein,4812, c -> t, S, NA
orf1.fasta,Replicase protein,4827, a -> t, N, K -> N
orf1.fasta,Replicase protein,4829, a -> m, N, Y -> X
orf1.fasta,Replicase protein,4830, t -> y, N, Y -> X
orf1.fasta,Replicase protein,4866, a -> t, S, NA
orf1.fasta,Replicase protein,4875, g -> t, N, E -> D
orf1.fasta,Replicase protein,4878, g -> a, S, NA
orf1.fasta,Replicase protein,4881, a -> g, S, NA
orf1.fasta,Replicase protein,4887, t -> a, S, NA
orf1.fasta,Replicase protein,4896, a -> t, S, NA
orf1.fasta,Replicase protein,4911, a -> g, S, NA
orf1.fasta,Replicase protein,4917, g -> a, S, NA
orf1.fasta,Replicase protein,4920, g -> a, S, NA
orf1.fasta,Replicase protein,4947, c -> t, S, NA
orf1.fasta,Replicase protein,4951, a -> t, N, N -> F
orf1.fasta,Replicase protein,4952, a -> t, N, N -> F
orf1.fasta,Replicase protein,4956, g -> a, S, NA
orf1.fasta,Replicase protein,4962, a -> g, S, NA
orf1.fasta,Replicase protein,4969, g -> t, N, V -> F
orf1.fasta,Replicase protein,4986, t -> c, S, NA
orf1.fasta,Replicase protein,4998, g -> a, S, NA

orf1.fasta,Replicase protein,5028, c -> t, S, NA
orf1.fasta,Replicase protein,5038, t -> c, S, NA
orf1.fasta,Replicase protein,5040, g -> c, S, NA
orf1.fasta,Replicase protein,5051, c -> a, N, T -> N
orf1.fasta,Replicase protein,5055, t -> g, S, NA
orf1.fasta,Replicase protein,5058, g -> a, S, NA
orf1.fasta,Replicase protein,5085, c -> g, S, NA
orf1.fasta,Replicase protein,5103, g -> t, S, NA
orf1.fasta,Replicase protein,5124, a -> g, S, NA
orf1.fasta,Replicase protein,5125, g -> t, N, A -> S
orf1.fasta,Replicase protein,5130, a -> g, S, NA
orf1.fasta,Replicase protein,5134, a -> t, N, M -> L
orf1.fasta,Replicase protein,5145, a -> t, S, NA
orf1.fasta,Replicase protein,5148, t -> g, S, NA
orf1.fasta,Replicase protein,5157, c -> t, S, NA
orf1.fasta,Replicase protein,5160, a -> t, S, NA
orf1.fasta,Replicase protein,5172, t -> c, S, NA
orf1.fasta,Replicase protein,5217, t -> a, S, NA
orf1.fasta,Replicase protein,5225, c -> t, N, T -> I
orf1.fasta,Replicase protein,5238, g -> a, S, NA
orf1.fasta,Replicase protein,5241, c -> t, S, NA
orf1.fasta,Replicase protein,5265, a -> g, S, NA
orf1.fasta,Replicase protein,5281, t -> g, N, L -> V
orf1.fasta,Replicase protein,5283, a -> t, N, L -> V
orf1.fasta,Replicase protein,5322, a -> t, N, E -> D
orf1.fasta,Replicase protein,5328, a -> g, S, NA
orf1.fasta,Replicase protein,5329, g -> a, N, V -> I
orf1.fasta,Replicase protein,5334, c -> t, S, NA
orf1.fasta,Replicase protein,5340, a -> c, S, NA
orf1.fasta,Replicase protein,5352, c -> t, S, NA
orf1.fasta,Replicase protein,5355, t -> c, S, NA
orf1.fasta,Replicase protein,5367, g -> a, S, NA
orf1.fasta,Replicase protein,5370, t -> c, S, NA
orf1.fasta,Replicase protein,5373, t -> c, S, NA
orf1.fasta,Replicase protein,5379, g -> a, S, NA
orf1.fasta,Replicase protein,5401, a -> c, N, I -> L
orf1.fasta,Replicase protein,5406, c -> t, S, NA
orf1.fasta,Replicase protein,5419, t -> g, N, S -> A
orf1.fasta,Replicase protein,5427, t -> a, S, NA
orf1.fasta,Replicase protein,5430, g -> a, S, NA
orf1.fasta,Replicase protein,5445, t -> a, S, NA
orf1.fasta,Replicase protein,5447, t -> g, N, V -> G
orf1.fasta,Replicase protein,5449, t -> a, N, C -> S
orf1.fasta,Replicase protein,5465, t -> a, N, I -> K
orf1.fasta,Replicase protein,5509, g -> t, N, A -> S
orf1.fasta,Replicase protein,5544, g -> a, S, NA
orf1.fasta,Replicase protein,5548, g -> a, N, A -> T
orf1.fasta,Replicase protein,5553, t -> c, S, NA
orf1.fasta,Replicase protein,5578, c -> t, S, NA
orf1.fasta,Replicase protein,5583, c -> t, S, NA

orf1.fasta,Replicase protein,5601, g -> a, S, NA
orf1.fasta,Replicase protein,5619, g -> a, S, NA
orf1.fasta,Replicase protein,5631, c -> t, S, NA
orf1.fasta,Replicase protein,5646, a -> g, S, NA
orf1.fasta,Replicase protein,5661, c -> a, N, N -> K
orf1.fasta,Replicase protein,5667, t -> c, S, NA
orf1.fasta,Replicase protein,5668, c -> t, S, NA
orf1.fasta,Replicase protein,5688, t -> c, S, NA
orf1.fasta,Replicase protein,5703, c -> t, S, NA
orf1.fasta,Replicase protein,5721, g -> a, S, NA
orf1.fasta,Replicase protein,5724, a -> c, S, NA
orf1.fasta,Replicase protein,5733, g -> a, S, NA
orf1.fasta,Replicase protein,5776, g -> a, N, V -> I
orf1.fasta,Replicase protein,5787, t -> c, S, NA
orf1.fasta,Replicase protein,5798, g -> t, N, S -> I
orf1.fasta,Replicase protein,5802, c -> t, S, NA
orf1.fasta,Replicase protein,5825, g -> a, N, S -> N
orf1.fasta,Replicase protein,5832, g -> a, S, NA
orf1.fasta,Replicase protein,5850, g -> c, N, K -> N
orf1.fasta,Replicase protein,5871, a -> t, S, NA
orf1.fasta,Replicase protein,5922, g -> a, S, NA
orf1.fasta,Replicase protein,5931, c -> t, S, NA
orf1.fasta,Replicase protein,5943, c -> t, S, NA
orf1.fasta,Replicase protein,5964, t -> c, S, NA
orf1.fasta,Replicase protein,5971, a -> c, N, K -> Q
orf1.fasta,Replicase protein,5976, a -> t, S, NA
orf1.fasta,Replicase protein,5979, a -> c, S, NA
orf1.fasta,Replicase protein,5982, c -> t, S, NA
orf1.fasta,Replicase protein,6003, c -> t, S, NA
orf1.fasta,Replicase protein,6011, c -> t, N, S -> L
orf1.fasta,Replicase protein,6013, c -> t, S, NA
orf1.fasta,Replicase protein,6018, c -> t, S, NA
orf1.fasta,Replicase protein,6024, t -> g, N, D -> E
orf1.fasta,Replicase protein,6035, c -> a, N, A -> D
orf1.fasta,Replicase protein,6039, g -> t, S, NA
orf1.fasta,Replicase protein,6048, a -> g, S, NA
orf1.fasta,Replicase protein,6050, c -> a, N, A -> D
orf1.fasta,Replicase protein,6061, a -> g, N, S -> D
orf1.fasta,Replicase protein,6062, g -> a, N, S -> D
orf1.fasta,Replicase protein,6068, a -> g, N, D -> G
orf1.fasta,Replicase protein,6074, g -> t, N, S -> I
orf1.fasta,Replicase protein,6078, t -> a, S, NA
orf1.fasta,Replicase protein,6086, c -> a, N, A -> D
orf1.fasta,Replicase protein,6089, t -> c, N, V -> A
orf1.fasta,Replicase protein,6097, a -> t, N, T -> S
orf1.fasta,Replicase protein,6099, a -> c, N, T -> S
orf1.fasta,Replicase protein,6103, a -> s, N, K -> X
orf1.fasta,Replicase protein,6106, a -> w, N, I -> X
orf1.fasta,Replicase protein,6107, t -> k, N, I -> X
orf1.fasta,Replicase protein,6108, c -> m, N, I -> X

orf1.fasta,Replicase protein,6109, a -> m, N, N -> X
orf1.fasta,Replicase protein,6112, a -> w, N, I -> X
orf1.fasta,Replicase protein,6113, t -> w, N, I -> X
orf1.fasta,Replicase protein,6114, c -> y, N, I -> X
orf1.fasta,Replicase protein,6115, g -> m, N, V -> X
orf1.fasta,Replicase protein,6118, c -> m, N, Q -> X
orf1.fasta,Replicase protein,6120, g -> r, N, Q -> X
orf1.fasta,Replicase protein,6125, a -> r, N, N -> X
orf1.fasta,Replicase protein,6130, g -> k, N, V -> X
orf1.fasta,Replicase protein,6138, g -> a, S, NA
orf1.fasta,Replicase protein,6155, a -> g, N, D -> G
orf1.fasta,Replicase protein,6163, g -> a, N, V -> I
orf1.fasta,Replicase protein,6166, a -> g, N, I -> V
orf1.fasta,Replicase protein,6177, c -> t, S, NA
orf1.fasta,Replicase protein,6183, c -> t, S, NA
orf1.fasta,Replicase protein,6186, g -> a, S, NA
orf1.fasta,Replicase protein,6188, c -> t, N, T -> I
orf1.fasta,Replicase protein,6204, c -> t, S, NA
orf1.fasta,Replicase protein,6213, t -> a, S, NA
orf1.fasta,Replicase protein,6248, a -> g, N, K -> R
orf1.fasta,Replicase protein,6260, a -> g, N, K -> R
orf1.fasta,Replicase protein,6261, a -> g, N, K -> R
orf1.fasta,Replicase protein,6281, g -> t, N, R -> M
orf1.fasta,Replicase protein,6291, t -> c, S, NA
orf1.fasta,Replicase protein,6311, a -> t, N, Y -> F
orf1.fasta,Replicase protein,6314, t -> b, N, I -> X
orf1.fasta,Replicase protein,6316, a -> h, N, K -> X
orf1.fasta,Replicase protein,6318, a -> h, N, K -> X
orf1.fasta,Replicase protein,6321, t -> h, N, F -> X
orf1.fasta,Replicase protein,6323, g -> v, N, G -> X
orf1.fasta,Replicase protein,6326, t -> c, N, M -> X
orf1.fasta,Replicase protein,6327, g -> v, N, M -> X
orf1.fasta,Replicase protein,6328, a -> d, N, T -> X
orf1.fasta,Replicase protein,6331, c -> s, N, L -> X
orf1.fasta,Replicase protein,6332, t -> w, N, L -> X
orf1.fasta,Replicase protein,6333, t -> c, N, L -> X
orf1.fasta,Replicase protein,6338, g -> v, N, S -> X
orf1.fasta,Replicase protein,6340, a -> d, N, I -> X
orf1.fasta,Replicase protein,6343, c -> v, N, P -> X
orf1.fasta,Replicase protein,6347, t -> a, N, I -> N
orf1.fasta,Replicase protein,6348, a -> t, N, I -> N
orf1.fasta,Replicase protein,6355, t -> k, N, L -> X
orf1.fasta,Replicase protein,6358, a -> w, N, N -> X
orf1.fasta,Replicase protein,6360, t -> y, N, N -> X
orf1.fasta,Replicase protein,6369, t -> g, N, D -> E
orf1.fasta,Replicase protein,6380, t -> c, N, V -> A
orf1.fasta,Replicase protein,6382, g -> t, N, V -> F
orf1.fasta,Replicase protein,6395, g -> a, N, R -> K
orf1.fasta,Replicase protein,6399, g -> t, S, NA
orf1.fasta,Replicase protein,6412, g -> a, N, V -> I

orf1.fasta,Replicase protein,6456, g -> a, S, NA
orf1.fasta,Replicase protein,6475, t -> c, N, S -> P
orf1.fasta,Replicase protein,6480, c -> t, S, NA
orf1.fasta,Replicase protein,6495, a -> t, S, NA
orf1.fasta,Replicase protein,6508, a -> g, N, I -> V
orf1.fasta,Replicase protein,6525, t -> a, S, NA
orf1.fasta,Replicase protein,6619, t -> c, S, NA
orf1.fasta,Replicase protein,6671, t -> y, N, L -> X
orf1.fasta,Replicase protein,6672, a -> r, N, L -> X
orf1.fasta,Replicase protein,6673, c -> m, N, P -> X
orf1.fasta,Replicase protein,6675, c -> w, N, P -> X
orf1.fasta,Replicase protein,6679, a -> r, N, I -> X
orf1.fasta,Replicase protein,6681, t -> c, N, I -> X
orf1.fasta,Replicase protein,6684, c -> y, N, G -> X
orf1.fasta,Replicase protein,6685, t -> w, N, F -> X
orf1.fasta,Replicase protein,6686, t -> w, N, F -> X
orf1.fasta,Replicase protein,6687, t -> s, N, F -> X
orf1.fasta,Replicase protein,6688, t -> a, N, L -> X
orf1.fasta,Replicase protein,6689, t -> w, N, L -> X
orf1.fasta,Replicase protein,6690, g -> d, N, L -> X
orf1.fasta,Replicase protein,6696, c -> t, S, NA
orf1.fasta,Replicase protein,6699, c -> t, S, NA
orf1.fasta,Replicase protein,6708, a -> g, S, NA
orf1.fasta,Replicase protein,6711, t -> c, S, NA
orf1.fasta,Replicase protein,6713, c -> g, N, A -> G
orf1.fasta,Replicase protein,6714, t -> a, N, A -> G
orf1.fasta,Replicase protein,6728, g -> a, N, S -> N
orf1.fasta,Replicase protein,6744, t -> a, S, NA
orf1.fasta,Replicase protein,6757, t -> c, N, F -> L
orf1.fasta,Replicase protein,6759, t -> a, N, F -> L
orf1.fasta,Replicase protein,6766, g -> a, N, V -> I
orf1.fasta,Replicase protein,6769, c -> t, N, Q -> Y
orf1.fasta,Replicase protein,6771, a -> t, N, Q -> Y
orf1.fasta,Replicase protein,6786, a -> g, S, NA
orf1.fasta,Replicase protein,6819, g -> a, S, NA
orf1.fasta,Replicase protein,6829, t -> g, N, S -> A
orf1.fasta,Replicase protein,6873, a -> g, S, NA
orf1.fasta,Replicase protein,6896, t -> c, N, V -> A
orf1.fasta,Replicase protein,6898, g -> t, N, V -> F
orf1.fasta,Replicase protein,6921, g -> a, S, NA
orf1.fasta,Replicase protein,6924, a -> g, S, NA
orf1.fasta,Replicase protein,6945, a -> t, S, NA
orf1.fasta,Replicase protein,6963, a -> g, S, NA
orf1.fasta,Replicase protein,6975, t -> c, S, NA
orf1.fasta,Replicase protein,6978, t -> a, S, NA
orf1.fasta,Replicase protein,6993, c -> t, S, NA
orf1.fasta,Replicase protein,6997, c -> a, N, L -> I
orf1.fasta,Replicase protein,7005, t -> c, S, NA
orf1.fasta,Replicase protein,7008, a -> g, S, NA
orf1.fasta,Replicase protein,7011, t -> c, S, NA

orf1.fasta,Replicase protein,7020, a -> g, S, NA
orf1.fasta,Replicase protein,7040, g -> a, N, S -> N
orf1.fasta,Replicase protein,7041, t -> c, N, S -> N
orf1.fasta,Replicase protein,7047, a -> g, S, NA
orf1.fasta,Replicase protein,7057, t -> g, N, F -> V
orf1.fasta,Replicase protein,7066, t -> c, S, NA
orf1.fasta,Replicase protein,7071, a -> g, S, NA
orf1.fasta,Replicase protein,7077, g -> a, S, NA
orf1.fasta,Replicase protein,7095, g -> a, S, NA
orf1.fasta,Replicase protein,7101, t -> g, S, NA
orf1.fasta,Replicase protein,7110, a -> g, S, NA
orf1.fasta,Replicase protein,7119, c -> t, S, NA
orf1.fasta,Replicase protein,7143, t -> g, S, NA
orf1.fasta,Replicase protein,7152, a -> g, S, NA
orf1.fasta,Replicase protein,7203, t -> c, S, NA
orf1.fasta,Replicase protein,7215, c -> t, S, NA
orf1.fasta,Replicase protein,7218, t -> c, S, NA
orf1.fasta,Replicase protein,7227, a -> t, S, NA
orf1.fasta,Replicase protein,7251, t -> c, S, NA
orf1.fasta,Replicase protein,7272, g -> t, S, NA
orf1.fasta,Replicase protein,7290, c -> t, S, NA
orf1.fasta,Replicase protein,7311, g -> a, S, NA
orf1.fasta,Replicase protein,7338, c -> t, S, NA
orf1.fasta,Replicase protein,7359, t -> c, S, NA
orf1.fasta,Replicase protein,7371, t -> c, S, NA
orf1.fasta,Replicase protein,7375, t -> c, N, F -> L
orf1.fasta,Replicase protein,7381, c -> t, S, NA
orf1.fasta,Replicase protein,7386, a -> t, S, NA
orf1.fasta,Replicase protein,7388, g -> a, N, R -> K
orf1.fasta,Replicase protein,7389, a -> g, N, R -> K
orf1.fasta,Replicase protein,7398, g -> a, S, NA
orf1.fasta,Replicase protein,7402, c -> t, N, P -> S
orf1.fasta,Replicase protein,7404, c -> t, N, P -> S
orf1.fasta,Replicase protein,7428, c -> t, S, NA
orf1.fasta,Replicase protein,7434, a -> g, S, NA
orf1.fasta,Replicase protein,7437, a -> t, S, NA
orf1.fasta,Replicase protein,7463, c -> a, N, S -> Y
orf1.fasta,Replicase protein,7470, a -> c, S, NA
orf1.fasta,Replicase protein,7471, c -> t, S, NA
orf1.fasta,Replicase protein,7479, c -> t, S, NA
orf1.fasta,Replicase protein,7482, t -> g, N, D -> E
orf1.fasta,Replicase protein,7485, c -> t, S, NA
orf1.fasta,Replicase protein,7491, g -> a, S, NA
orf1.fasta,Replicase protein,7500, t -> a, S, NA
orf1.fasta,Replicase protein,7543, c -> t, S, NA
orf1.fasta,Replicase protein,7564, a -> g, N, S -> G
orf1.fasta,Replicase protein,7590, t -> a, S, NA
orf1.fasta,Replicase protein,7593, a -> g, S, NA
orf1.fasta,Replicase protein,7605, t -> c, S, NA
orf1.fasta,Replicase protein,7623, g -> t, S, NA

orf1.fasta,Replicase protein,7647, a -> g, S, NA
orf1.fasta,Replicase protein,7650, t -> c, S, NA
orf1.fasta,Replicase protein,7698, c -> t, S, NA
orf1.fasta,Replicase protein,7746, t -> c, S, NA
orf1.fasta,Replicase protein,7749, c -> a, S, NA
orf1.fasta,Replicase protein,7770, a -> g, S, NA
orf1.fasta,Replicase protein,7799, t -> c, N, V -> A
orf1.fasta,Replicase protein,7818, t -> a, S, NA
orf1.fasta,Replicase protein,7837, g -> t, N, D -> C
orf1.fasta,Replicase protein,7838, a -> g, N, D -> C
orf1.fasta,Replicase protein,7851, c -> t, S, NA
orf1.fasta,Replicase protein,7875, g -> a, S, NA
orf1.fasta,Replicase protein,7901, g -> a, N, G -> D
orf1.fasta,Replicase protein,7905, c -> t, S, NA
orf1.fasta,Replicase protein,7931, c -> m, N, S -> X
orf1.fasta,Replicase protein,7938, g -> a, S, NA
orf1.fasta,Replicase protein,7941, g -> t, S, NA
orf1.fasta,Replicase protein,7942, g -> t, N, A -> X
orf1.fasta,Replicase protein,7944, a -> w, N, A -> X
orf1.fasta,Replicase protein,7946, g -> t, N, G -> X
orf1.fasta,Replicase protein,7947, t -> s, N, G -> X
orf1.fasta,Replicase protein,7950, t -> k, N, L -> X
orf1.fasta,Replicase protein,7954, t -> a, N, L -> T
orf1.fasta,Replicase protein,7955, t -> c, N, L -> T
orf1.fasta,Replicase protein,7962, t -> d, N, D -> X
orf1.fasta,Replicase protein,7965, a -> h, N, E -> X
orf1.fasta,Replicase protein,7971, t -> h, N, C -> X
orf1.fasta,Replicase protein,7973, a -> g, N, N -> S
orf1.fasta,Replicase protein,7976, a -> v, N, N -> X
orf1.fasta,Replicase protein,7977, t -> a, N, N -> X
orf1.fasta,Replicase protein,7978, t -> b, N, L -> X
orf1.fasta,Replicase protein,7979, t -> g, N, L -> X
orf1.fasta,Replicase protein,7981, g -> t, N, V -> X
orf1.fasta,Replicase protein,7982, t -> h, N, V -> X
orf1.fasta,Replicase protein,7984, c -> t, N, P -> C
orf1.fasta,Replicase protein,7985, c -> g, N, P -> C
orf1.fasta,Replicase protein,7987, a -> d, N, T -> X
orf1.fasta,Replicase protein,7988, c -> s, N, T -> X
orf1.fasta,Replicase protein,8007, c -> t, S, NA
orf1.fasta,Replicase protein,8013, g -> t, S, NA
orf1.fasta,Replicase protein,8037, t -> a, S, NA
orf1.fasta,Replicase protein,8064, c -> t, S, NA
orf1.fasta,Replicase protein,8082, t -> c, S, NA
orf1.fasta,Replicase protein,8091, t -> c, S, NA
orf1.fasta,Replicase protein,8094, c -> t, S, NA
orf1.fasta,Replicase protein,8106, t -> g, S, NA
orf1.fasta,Replicase protein,8136, t -> c, S, NA
orf1.fasta,Replicase protein,8154, g -> a, S, NA
orf1.fasta,Replicase protein,8175, a -> g, S, NA
orf1.fasta,Replicase protein,8179, t -> c, S, NA

orf1.fasta,Replicase protein,8181, g -> c, S, NA
orf1.fasta,Replicase protein,8221, c -> t, S, NA
orf1.fasta,Replicase protein,8232, t -> c, S, NA
orf1.fasta,Replicase protein,8235, c -> t, S, NA
orf1.fasta,Replicase protein,8244, g -> a, S, NA
orf1.fasta,Replicase protein,8247, a -> g, S, NA
orf1.fasta,Replicase protein,8256, t -> a, S, NA
orf1.fasta,Replicase protein,8271, g -> t, S, NA
orf1.fasta,Replicase protein,8277, g -> a, S, NA
orf1.fasta,Replicase protein,8296, a -> g, N, I -> V
orf1.fasta,Replicase protein,8307, t -> a, S, NA
orf1.fasta,Replicase protein,8310, g -> a, S, NA
orf1.fasta,Replicase protein,8319, c -> t, S, NA
orf1.fasta,Replicase protein,8328, t -> c, S, NA
orf1.fasta,Replicase protein,8334, t -> c, S, NA
orf1.fasta,Replicase protein,8335, g -> a, N, A -> I
orf1.fasta,Replicase protein,8336, c -> t, N, A -> I
orf1.fasta,Replicase protein,8341, t -> c, N, Y -> H
orf1.fasta,Replicase protein,8385, g -> t, S, NA
orf1.fasta,Replicase protein,8410, a -> g, N, I -> V
orf1.fasta,Replicase protein,8430, c -> t, S, NA
orf1.fasta,Replicase protein,8454, c -> t, S, NA
orf1.fasta,Replicase protein,8472, c -> t, S, NA
orf1.fasta,Replicase protein,8475, g -> a, S, NA
orf1.fasta,Replicase protein,8486, a -> g, N, N -> S
orf1.fasta,Replicase protein,8496, c -> t, S, NA
orf1.fasta,Replicase protein,8501, a -> g, N, N -> S
orf1.fasta,Replicase protein,8529, a -> t, S, NA
orf1.fasta,Replicase protein,8530, a -> g, N, I -> V
orf1.fasta,Replicase protein,8542, a -> c, N, I -> L
orf1.fasta,Replicase protein,8556, g -> t, S, NA
orf1.fasta,Replicase protein,8577, t -> g, S, NA
orf1.fasta,Replicase protein,8604, t -> c, S, NA
orf1.fasta,Replicase protein,8610, c -> t, S, NA
orf1.fasta,Replicase protein,8613, g -> a, S, NA
orf1.fasta,Replicase protein,8620, t -> c, N, F -> L
orf1.fasta,Replicase protein,8622, c -> t, N, F -> L
orf1.fasta,Replicase protein,8625, c -> t, S, NA
orf1.fasta,Replicase protein,8649, t -> g, S, NA
orf1.fasta,Replicase protein,8673, c -> t, S, NA
orf1.fasta,Replicase protein,8675, c -> a, N, T -> N
orf1.fasta,Replicase protein,8706, t -> c, S, NA
orf1.fasta,Replicase protein,8709, c -> t, S, NA
orf1.fasta,Replicase protein,8712, c -> t, S, NA
orf1.fasta,Replicase protein,8715, c -> t, S, NA
orf1.fasta,Replicase protein,8730, t -> c, S, NA
orf1.fasta,Replicase protein,8737, c -> a, N, R -> S
orf1.fasta,Replicase protein,8739, c -> t, N, R -> S
orf1.fasta,Replicase protein,8763, a -> g, S, NA
orf1.fasta,Replicase protein,8775, a -> g, S, NA

orf1.fasta,Replicase protein,8778, t -> c, S, NA
orf1.fasta,Replicase protein,8802, g -> a, S, NA
orf1.fasta,Replicase protein,8808, c -> t, S, NA
orf1.fasta,Replicase protein,8820, c -> t, S, NA
orf1.fasta,Replicase protein,8860, c -> t, S, NA
orf1.fasta,Replicase protein,8871, g -> a, S, NA
orf1.fasta,Replicase protein,8886, t -> g, S, NA
orf1.fasta,Replicase protein,8892, a -> t, S, NA
orf1.fasta,Replicase protein,8919, t -> a, S, NA
orf1.fasta,Replicase protein,8920, c -> t, S, NA
orf1.fasta,Replicase protein,8931, t -> a, N, D -> E
orf1.fasta,Replicase protein,8944, g -> a, N, V -> I
orf1.fasta,Replicase protein,8946, c -> a, N, V -> I
orf1.fasta,Replicase protein,8949, t -> c, S, NA
orf1.fasta,Replicase protein,8967, c -> g, S, NA
orf1.fasta,Replicase protein,8973, a -> g, S, NA
orf1.fasta,Replicase protein,8985, g -> t, N, E -> D
orf1.fasta,Replicase protein,9039, c -> t, S, NA
orf1.fasta,Replicase protein,9048, g -> t, S, NA
orf1.fasta,Replicase protein,9078, t -> c, S, NA
orf1.fasta,Replicase protein,9079, c -> t, S, NA
orf1.fasta,Replicase protein,9090, t -> a, S, NA
orf1.fasta,Replicase protein,9099, c -> t, S, NA
orf1.fasta,Replicase protein,9129, c -> t, S, NA
orf1.fasta,Replicase protein,9148, c -> t, S, NA
orf1.fasta,Replicase protein,9187, g -> a, N, V -> I
orf1.fasta,Replicase protein,9189, g -> t, N, V -> I
orf1.fasta,Replicase protein,9205, g -> a, N, V -> I
orf1.fasta,Replicase protein,9207, a -> t, N, V -> I
orf1.fasta,Replicase protein,9219, a -> g, S, NA
orf1.fasta,Replicase protein,9240, a -> g, S, NA
orf1.fasta,Replicase protein,9311, a -> c, N, Y -> S
orf1.fasta,Replicase protein,9312, t -> a, N, Y -> S
orf1.fasta,Replicase protein,9403, t -> g, N, F -> V
orf1.fasta,Replicase protein,9418, c -> t, N, L -> F
orf1.fasta,Replicase protein,9420, c -> t, N, L -> F
orf1.fasta,Replicase protein,9430, t -> g, N, S -> A
orf1.fasta,Replicase protein,9443, a -> g, N, Q -> R
orf1.fasta,Replicase protein,9471, c -> t, S, NA
orf1.fasta,Replicase protein,9492, c -> t, S, NA
orf1.fasta,Replicase protein,9498, g -> t, S, NA
orf1.fasta,Replicase protein,9543, a -> g, S, NA
orf1.fasta,Replicase protein,9575, t -> a, N, L -> X
orf1.fasta,Replicase protein,9576, g -> s, N, L -> X
orf1.fasta,Replicase protein,9577, t -> a, N, Y -> X
orf1.fasta,Replicase protein,9578, a -> v, N, Y -> X
orf1.fasta,Replicase protein,9582, t -> b, N, N -> X
orf1.fasta,Replicase protein,9583, a -> m, N, K -> X
orf1.fasta,Replicase protein,9600, t -> c, S, NA
orf1.fasta,Replicase protein,9627, a -> g, S, NA

orf1.fasta,Replicase protein,9630, a -> g, S, NA
orf1.fasta,Replicase protein,9704, c -> h, N, P -> X
orf1.fasta,Replicase protein,9705, a -> d, N, P -> X
orf1.fasta,Replicase protein,9708, c -> t, S, NA
orf1.fasta,Replicase protein,9709, a -> h, N, T -> X
orf1.fasta,Replicase protein,9713, c -> y, N, A -> X
orf1.fasta,Replicase protein,9717, t -> c, S, NA
orf1.fasta,Replicase protein,9720, g -> t, S, NA
orf1.fasta,Replicase protein,9732, t -> c, S, NA
orf1.fasta,Replicase protein,9741, a -> t, S, NA
orf1.fasta,Replicase protein,9759, t -> k, N, V -> X
orf1.fasta,Replicase protein,9760, a -> w, N, N -> X
orf1.fasta,Replicase protein,9763, c -> m, N, P -> X
orf1.fasta,Replicase protein,9767, c -> s, N, T -> X
orf1.fasta,Replicase protein,9768, t -> w, N, T -> X
orf1.fasta,Replicase protein,9770, c -> m, N, S -> X
orf1.fasta,Replicase protein,9777, g -> a, S, NA
orf1.fasta,Replicase protein,9792, t -> c, S, NA
orf1.fasta,Replicase protein,9801, a -> c, S, NA
orf1.fasta,Replicase protein,9809, c -> a, N, T -> N
orf1.fasta,Replicase protein,9855, a -> t, S, NA
orf1.fasta,Replicase protein,9863, t -> k, N, V -> X
orf1.fasta,Replicase protein,9896, a -> c, N, D -> A
orf1.fasta,Replicase protein,9912, a -> g, S, NA
orf1.fasta,Replicase protein,9978, c -> t, S, NA
orf1.fasta,Replicase protein,10002, g -> t, S, NA
orf1.fasta,Replicase protein,10008, t -> a, S, NA
orf1.fasta,Replicase protein,10015, t -> c, S, NA
orf1.fasta,Replicase protein,10023, t -> c, S, NA
orf1.fasta,Replicase protein,10032, a -> g, S, NA
orf1.fasta,Replicase protein,10053, t -> g, S, NA
orf1.fasta,Replicase protein,10059, g -> a, S, NA
orf1.fasta,Replicase protein,10122, t -> g, S, NA
orf1.fasta,Replicase protein,10143, t -> c, S, NA
orf1.fasta,Replicase protein,10176, g -> a, S, NA
orf1.fasta,Replicase protein,10191, a -> t, S, NA
orf1.fasta,Replicase protein,10192, a -> c, N, I -> L
orf1.fasta,Replicase protein,10242, c -> t, S, NA
orf1.fasta,Replicase protein,10266, c -> t, S, NA
orf1.fasta,Replicase protein,10272, c -> t, S, NA
orf1.fasta,Replicase protein,10293, c -> t, S, NA
orf1.fasta,Replicase protein,10296, a -> g, S, NA
orf1.fasta,Replicase protein,10362, c -> t, S, NA
orf1.fasta,Replicase protein,10375, c -> t, N, L -> F
orf1.fasta,Replicase protein,10380, c -> t, S, NA
orf1.fasta,Replicase protein,10443, a -> g, S, NA
orf1.fasta,Replicase protein,10446, c -> t, S, NA
orf1.fasta,Replicase protein,10452, a -> g, S, NA
orf1.fasta,Replicase protein,10462, t -> g, N, F -> V
orf1.fasta,Replicase protein,10491, g -> a, S, NA

orf1.fasta,Replicase protein,10503, g -> t, S, NA
orf1.fasta,Replicase protein,10512, a -> t, S, NA
orf1.fasta,Replicase protein,10524, c -> a, S, NA
orf1.fasta,Replicase protein,10530, c -> t, S, NA
orf1.fasta,Replicase protein,10557, c -> t, S, NA
orf1.fasta,Replicase protein,10563, g -> a, S, NA
orf1.fasta,Replicase protein,10611, c -> t, S, NA
orf1.fasta,Replicase protein,10617, c -> t, S, NA
orf1.fasta,Replicase protein,10626, g -> a, S, NA
orf1.fasta,Replicase protein,10656, c -> t, S, NA
orf1.fasta,Replicase protein,10666, t -> g, N, F -> V
orf1.fasta,Replicase protein,10716, c -> t, S, NA
orf1.fasta,Replicase protein,10732, g -> s, N, V -> X
orf1.fasta,Replicase protein,10734, a -> g, N, V -> X
orf1.fasta,Replicase protein,10751, t -> k, N, M -> X
orf1.fasta,Replicase protein,10753, t -> w, N, Y -> X
orf1.fasta,Replicase protein,10767, c -> t, S, NA
orf1.fasta,Replicase protein,10782, a -> g, S, NA
orf1.fasta,Replicase protein,10809, a -> g, S, NA
orf1.fasta,Replicase protein,10827, g -> a, S, NA
orf1.fasta,Replicase protein,10875, t -> a, S, NA
orf1.fasta,Replicase protein,10876, t -> c, S, NA
orf1.fasta,Replicase protein,10887, t -> c, S, NA
orf1.fasta,Replicase protein,10902, a -> g, S, NA
orf1.fasta,Replicase protein,10905, t -> c, S, NA
orf1.fasta,Replicase protein,10911, t -> g, N, H -> Q
orf1.fasta,Replicase protein,10945, c -> t, N, H -> Y
orf1.fasta,Replicase protein,10949, t -> a, N, F -> Y
orf1.fasta,Replicase protein,10953, c -> t, S, NA
orf1.fasta,Replicase protein,10974, t -> c, S, NA
orf1.fasta,Replicase protein,10979, a -> c, N, D -> A
orf1.fasta,Replicase protein,10983, a -> t, N, E -> D
orf1.fasta,Replicase protein,11008, g -> a, N, V -> I
orf1.fasta,Replicase protein,11019, t -> c, S, NA
orf1.fasta,Replicase protein,11055, t -> c, S, NA
orf1.fasta,Replicase protein,11065, a -> g, N, M -> V
orf1.fasta,Replicase protein,11076, c -> t, S, NA
orf1.fasta,Replicase protein,11082, t -> g, S, NA
orf1.fasta,Replicase protein,11097, a -> t, S, NA
orf1.fasta,Replicase protein,11112, c -> t, S, NA
orf1.fasta,Replicase protein,11115, c -> t, S, NA
orf1.fasta,Replicase protein,11119, a -> t, N, I -> L
orf1.fasta,Replicase protein,11124, a -> g, S, NA
orf1.fasta,Replicase protein,11145, a -> g, S, NA
orf1.fasta,Replicase protein,11151, c -> t, S, NA
orf1.fasta,Replicase protein,11182, c -> t, S, NA
orf1.fasta,Replicase protein,11184, t -> a, S, NA
orf1.fasta,Replicase protein,11187, c -> t, S, NA
orf1.fasta,Replicase protein,11196, t -> a, S, NA
orf1.fasta,Replicase protein,11202, g -> a, S, NA

orf1.fasta,Replicase protein,11253, c -> t, S, NA
orf1.fasta,Replicase protein,11262, a -> g, S, NA
orf1.fasta,Replicase protein,11272, t -> g, N, L -> V
orf1.fasta,Replicase protein,11274, g -> a, N, L -> V
orf1.fasta,Replicase protein,11289, t -> a, S, NA
orf1.fasta,Replicase protein,11316, a -> t, S, NA
orf1.fasta,Replicase protein,11331, g -> t, N, M -> I
orf1.fasta,Replicase protein,11334, t -> c, S, NA
orf1.fasta,Replicase protein,11352, c -> t, S, NA
orf1.fasta,Replicase protein,11376, a -> t, S, NA
orf1.fasta,Replicase protein,11424, c -> t, S, NA
orf1.fasta,Replicase protein,11463, g -> t, S, NA
orf1.fasta,Replicase protein,11484, a -> w, N, P -> X
orf1.fasta,Replicase protein,11486, t -> w, N, I -> X
orf1.fasta,Replicase protein,11490, t -> k, N, I -> X
orf1.fasta,Replicase protein,11491, g -> s, N, E -> X
orf1.fasta,Replicase protein,11493, a -> w, N, E -> X
orf1.fasta,Replicase protein,11496, a -> w, N, V -> X
orf1.fasta,Replicase protein,11497, t -> k, N, S -> X
orf1.fasta,Replicase protein,11498, c -> y, N, S -> X
orf1.fasta,Replicase protein,11499, t -> k, N, S -> X
orf1.fasta,Replicase protein,11500, c -> s, N, Q -> X
orf1.fasta,Replicase protein,11501, a -> w, N, Q -> X
orf1.fasta,Replicase protein,11503, t -> k, N, F -> X
orf1.fasta,Replicase protein,11505, t -> k, N, F -> X
orf1.fasta,Replicase protein,11506, c -> s, N, Q -> X
orf1.fasta,Replicase protein,11509, t -> w, N, S -> X
orf1.fasta,Replicase protein,11510, c -> m, N, S -> X
orf1.fasta,Replicase protein,11511, a -> w, N, S -> X
orf1.fasta,Replicase protein,11512, a -> r, N, K -> X
orf1.fasta,Replicase protein,11514, a -> w, N, K -> X
orf1.fasta,Replicase protein,11515, t -> k, N, L -> X
orf1.fasta,Replicase protein,11516, t -> y, N, L -> X
orf1.fasta,Replicase protein,11517, g -> s, N, L -> X
orf1.fasta,Replicase protein,11518, a -> r, N, T -> X
orf1.fasta,Replicase protein,11519, c -> m, N, T -> X
orf1.fasta,Replicase protein,11521, g -> r, N, D -> X
orf1.fasta,Replicase protein,11523, t -> w, N, D -> X
orf1.fasta,Replicase protein,11525, t -> y, N, V -> X
orf1.fasta,Replicase protein,11526, c -> t, N, V -> X
orf1.fasta,Replicase protein,11529, a -> w, N, K -> X
orf1.fasta,Replicase protein,11531, g -> k, N, C -> X
orf1.fasta,Replicase protein,11533, g -> s, N, A -> X
orf1.fasta,Replicase protein,11534, c -> y, N, A -> X
orf1.fasta,Replicase protein,11535, t -> y, N, A -> X
orf1.fasta,Replicase protein,11540, t -> c, N, V -> A
orf1.fasta,Replicase protein,11541, c -> t, N, V -> A
orf1.fasta,Replicase protein,11543, t -> y, N, V -> X
orf1.fasta,Replicase protein,11544, c -> t, N, V -> X
orf1.fasta,Replicase protein,11545, t -> k, N, L -> X

orf1.fasta,Replicase protein,11547, g -> r, N, L -> X
orf1.fasta,Replicase protein,11548, c -> y, N, L -> X
orf1.fasta,Replicase protein,11583, t -> c, S, NA
orf1.fasta,Replicase protein,11610, t -> g, S, NA
orf1.fasta,Replicase protein,11644, a -> g, N, S -> G
orf1.fasta,Replicase protein,11745, c -> t, S, NA
orf1.fasta,Replicase protein,11850, g -> t, S, NA
orf1.fasta,Replicase protein,12072, a -> g, S, NA
orf1.fasta,Replicase protein,12105, c -> t, S, NA
orf1.fasta,Replicase protein,12150, a -> t, S, NA
orf1.fasta,Replicase protein,12156, t -> c, S, NA
orf1.fasta,Replicase protein,12207, t -> c, S, NA
orf1.fasta,Replicase protein,12222, t -> c, S, NA
orf1.fasta,Replicase protein,12244, g -> a, N, V -> I
orf1.fasta,Replicase protein,12255, c -> t, S, NA
orf1.fasta,Replicase protein,12390, a -> g, S, NA
orf1.fasta,Replicase protein,12446, t -> a, N, L -> Q
orf1.fasta,Replicase protein,12465, t -> y, N, S -> X
orf1.fasta,Replicase protein,12471, t -> y, N, N -> X
orf1.fasta,Replicase protein,12472, g -> k, N, G -> X
orf1.fasta,Replicase protein,12476, a -> c, N, K -> T
orf1.fasta,Replicase protein,12483, t -> k, N, V -> X
orf1.fasta,Replicase protein,12525, a -> g, S, NA
orf1.fasta,Replicase protein,12555, c -> t, S, NA
orf1.fasta,Replicase protein,12714, a -> g, S, NA
orf1.fasta,Replicase protein,12738, g -> a, S, NA
orf1.fasta,Replicase protein,12792, a -> g, S, NA
orf1.fasta,Replicase protein,12795, a -> g, S, NA
orf1.fasta,Replicase protein,12812, t -> c, N, V -> A
orf1.fasta,Replicase protein,12813, c -> t, N, V -> A
orf1.fasta,Replicase protein,12866, c -> t, N, A -> V
orf1.fasta,Replicase protein,12873, c -> t, S, NA
orf1.fasta,Replicase protein,12887, g -> a, N, S -> N
orf1.fasta,Replicase protein,12980, g -> a, N, R -> K
orf1.fasta,Replicase protein,13040, t -> a, N, V -> D
orf1.fasta,Replicase protein,13071, c -> a, S, NA
orf1.fasta,Replicase protein,13104, t -> c, S, NA
orf1.fasta,Replicase protein,13107, a -> g, S, NA
orf1.fasta,Replicase protein,13266, t -> c, S, NA
orf1.fasta,Replicase protein,13299, c -> t, S, NA
orf1.fasta,Replicase protein,13446, c -> t, S, NA
orf1.fasta,Replicase protein,13458, g -> a, S, NA
orf1.fasta,Replicase protein,13485, t -> c, S, NA
orf1.fasta,Replicase protein,13545, c -> t, S, NA
orf1.fasta,Replicase protein,13557, t -> c, S, NA
orf1.fasta,Replicase protein,13583, a -> w, N, K -> X
orf1.fasta,Replicase protein,13584, g -> s, N, K -> X
orf1.fasta,Replicase protein,13585, a -> w, N, K -> X
orf1.fasta,Replicase protein,13586, a -> t, N, K -> X
orf1.fasta,Replicase protein,13590, t -> y, N, D -> X

orf1.fasta,Replicase protein,13591, t -> w, N, W -> X
orf1.fasta,Replicase protein,13599, t -> y, N, D -> X
orf1.fasta,Replicase protein,13627, t -> g, N, L -> V
orf1.fasta,Replicase protein,13629, g -> t, N, L -> V
orf1.fasta,Replicase protein,13641, g -> a, S, NA
orf1.fasta,Replicase protein,13651, c -> t, N, L -> F
orf1.fasta,Replicase protein,13656, t -> w, N, N -> X
orf1.fasta,Replicase protein,13659, a -> w, N, R -> X
orf1.fasta,Replicase protein,13680, g -> k, N, E -> X
orf1.fasta,Replicase protein,13686, g -> a, S, NA
orf1.fasta,Replicase protein,13694, t -> c, N, L -> S
orf1.fasta,Replicase protein,13699, g -> r, N, E -> X
orf1.fasta,Replicase protein,13700, a -> w, N, E -> X
orf1.fasta,Replicase protein,13710, a -> g, S, NA
orf1.fasta,Replicase protein,13717, g -> a, N, V -> I
orf1.fasta,Replicase protein,13734, t -> c, S, NA
orf1.fasta,Replicase protein,13791, a -> t, S, NA
orf1.fasta,Replicase protein,13812, t -> c, S, NA
orf1.fasta,Replicase protein,13842, t -> g, S, NA
orf1.fasta,Replicase protein,13902, c -> t, S, NA
orf1.fasta,Replicase protein,13935, t -> c, S, NA
orf1.fasta,Replicase protein,13989, t -> g, S, NA
orf1.fasta,Replicase protein,14109, g -> t, S, NA
orf1.fasta,Replicase protein,14130, c -> a, S, NA
orf1.fasta,Replicase protein,14157, t -> g, S, NA
orf1.fasta,Replicase protein,14197, c -> t, S, NA
orf1.fasta,Replicase protein,14259, a -> g, S, NA
orf1.fasta,Replicase protein,14377, a -> c, N, M -> L
orf1.fasta,Replicase protein,14421, t -> c, S, NA
orf1.fasta,Replicase protein,14469, t -> c, S, NA
orf1.fasta,Replicase protein,14481, g -> c, S, NA
orf1.fasta,Replicase protein,14490, c -> t, S, NA
orf1.fasta,Replicase protein,14526, c -> t, S, NA
orf1.fasta,Replicase protein,14535, c -> t, S, NA
orf1.fasta,Replicase protein,14574, t -> c, S, NA
orf1.fasta,Replicase protein,14583, c -> t, S, NA
orf1.fasta,Replicase protein,14682, t -> c, S, NA
orf1.fasta,Replicase protein,14733, c -> t, S, NA
orf1.fasta,Replicase protein,14757, t -> c, S, NA
orf1.fasta,Replicase protein,14856, g -> t, S, NA
orf1.fasta,Replicase protein,14874, a -> g, S, NA
orf1.fasta,Replicase protein,14883, a -> c, S, NA
orf1.fasta,Replicase protein,14904, c -> t, S, NA
orf1.fasta,Replicase protein,14955, a -> t, S, NA
orf1.fasta,Replicase protein,15006, a -> t, S, NA
orf1.fasta,Replicase protein,15012, c -> t, S, NA
orf1.fasta,Replicase protein,15024, g -> a, S, NA
orf1.fasta,Replicase protein,15082, c -> t, S, NA
orf1.fasta,Replicase protein,15084, t -> g, S, NA
orf1.fasta,Replicase protein,15174, c -> t, S, NA

orf1.fasta,Replicase protein,15211, t -> g, N, S -> A
orf1.fasta,Replicase protein,15213, c -> t, N, S -> A
orf1.fasta,Replicase protein,15231, c -> t, S, NA
orf1.fasta,Replicase protein,15273, t -> c, S, NA
orf1.fasta,Replicase protein,15381, t -> c, S, NA
orf1.fasta,Replicase protein,15393, t -> c, S, NA
orf1.fasta,Replicase protein,15453, a -> g, S, NA
orf1.fasta,Replicase protein,15516, a -> t, N, K -> N
orf1.fasta,Replicase protein,15537, g -> a, S, NA
orf1.fasta,Replicase protein,15552, a -> t, S, NA
orf1.fasta,Replicase protein,15567, a -> g, S, NA
orf1.fasta,Replicase protein,15579, c -> t, S, NA
orf1.fasta,Replicase protein,15603, c -> t, S, NA
orf1.fasta,Replicase protein,15621, c -> t, S, NA
orf1.fasta,Replicase protein,15627, g -> a, S, NA
orf1.fasta,Replicase protein,15708, t -> c, S, NA
orf1.fasta,Replicase protein,15780, a -> g, S, NA
orf1.fasta,Replicase protein,15810, c -> t, S, NA
orf1.fasta,Replicase protein,15837, a -> g, S, NA
orf1.fasta,Replicase protein,15846, a -> t, N, E -> D
orf1.fasta,Replicase protein,15888, g -> r, N, M -> X
orf1.fasta,Replicase protein,15897, c -> t, S, NA
orf1.fasta,Replicase protein,15904, t -> b, N, C -> X
orf1.fasta,Replicase protein,15905, g -> r, N, C -> X
orf1.fasta,Replicase protein,15908, t -> w, N, V -> X
orf1.fasta,Replicase protein,15911, t -> w, N, V -> X
orf1.fasta,Replicase protein,15912, t -> m, N, V -> X
orf1.fasta,Replicase protein,15913, t -> s, N, C -> X
orf1.fasta,Replicase protein,15917, c -> g, N, S -> C
orf1.fasta,Replicase protein,15919, t -> a, N, S -> X
orf1.fasta,Replicase protein,15920, c -> t, N, S -> X
orf1.fasta,Replicase protein,15921, t -> d, N, S -> X
orf1.fasta,Replicase protein,15923, a -> w, N, Q -> X
orf1.fasta,Replicase protein,15925, a -> v, N, T -> X
orf1.fasta,Replicase protein,15926, c -> t, N, T -> X
orf1.fasta,Replicase protein,15928, t -> g, N, S -> X
orf1.fasta,Replicase protein,15929, c -> s, N, S -> X
orf1.fasta,Replicase protein,15932, t -> g, N, L -> X
orf1.fasta,Replicase protein,15933, a -> r, N, L -> X
orf1.fasta,Replicase protein,15937, t -> w, N, C -> X
orf1.fasta,Replicase protein,15942, c -> a, N, G -> X
orf1.fasta,Replicase protein,15945, t -> g, N, S -> R
orf1.fasta,Replicase protein,15948, c -> m, N, C -> X
orf1.fasta,Replicase protein,15949, a -> r, N, I -> X
orf1.fasta,Replicase protein,15950, t -> k, N, I -> X
orf1.fasta,Replicase protein,15951, c -> m, N, I -> X
orf1.fasta,Replicase protein,15954, a -> w, N, R -> X
orf1.fasta,Replicase protein,15955, a -> r, N, K -> X
orf1.fasta,Replicase protein,15956, a -> w, N, K -> X
orf1.fasta,Replicase protein,15957, g -> r, N, K -> X

orf1.fasta,Replicase protein,15958, c -> s, N, P -> X
orf1.fasta,Replicase protein,15990, t -> c, S, NA
orf1.fasta,Replicase protein,16076, g -> a, N, R -> K
orf1.fasta,Replicase protein,16113, c -> t, S, NA
orf1.fasta,Replicase protein,16131, a -> g, S, NA
orf1.fasta,Replicase protein,16134, c -> t, S, NA
orf1.fasta,Replicase protein,16185, t -> a, S, NA
orf1.fasta,Replicase protein,16191, a -> t, S, NA
orf1.fasta,Replicase protein,16194, g -> t, S, NA
orf1.fasta,Replicase protein,16200, t -> a, S, NA
orf1.fasta,Replicase protein,16215, c -> t, S, NA
orf1.fasta,Replicase protein,16218, t -> a, S, NA
orf1.fasta,Replicase protein,16221, c -> t, S, NA
orf1.fasta,Replicase protein,16236, c -> t, S, NA
orf1.fasta,Replicase protein,16242, g -> t, S, NA
orf1.fasta,Replicase protein,16255, t -> c, S, NA
orf1.fasta,Replicase protein,16260, a -> t, S, NA
orf1.fasta,Replicase protein,16302, t -> g, S, NA
orf1.fasta,Replicase protein,16311, a -> g, S, NA
orf1.fasta,Replicase protein,16320, a -> g, S, NA
orf1.fasta,Replicase protein,16359, a -> g, S, NA
orf1.fasta,Replicase protein,16368, t -> c, S, NA
orf1.fasta,Replicase protein,16386, c -> t, S, NA
orf1.fasta,Replicase protein,16413, g -> a, S, NA
orf1.fasta,Replicase protein,16416, a -> t, S, NA
orf1.fasta,Replicase protein,16425, g -> a, S, NA
orf1.fasta,Replicase protein,16434, g -> t, S, NA
orf1.fasta,Replicase protein,16473, c -> t, S, NA
orf1.fasta,Replicase protein,16482, a -> g, S, NA
orf1.fasta,Replicase protein,16497, a -> g, S, NA
orf1.fasta,Replicase protein,16518, g -> t, S, NA
orf1.fasta,Replicase protein,16524, c -> t, S, NA
orf1.fasta,Replicase protein,16557, t -> a, S, NA
orf1.fasta,Replicase protein,16569, g -> t, S, NA
orf1.fasta,Replicase protein,16575, a -> c, S, NA
orf1.fasta,Replicase protein,16578, g -> t, S, NA
orf1.fasta,Replicase protein,16581, c -> t, S, NA
orf1.fasta,Replicase protein,16584, g -> a, S, NA
orf1.fasta,Replicase protein,16614, c -> g, S, NA
orf1.fasta,Replicase protein,16617, a -> g, S, NA
orf1.fasta,Replicase protein,16635, c -> t, S, NA
orf1.fasta,Replicase protein,16680, c -> t, S, NA
orf1.fasta,Replicase protein,16686, t -> c, S, NA
orf1.fasta,Replicase protein,16701, t -> c, S, NA
orf1.fasta,Replicase protein,16707, a -> t, S, NA
orf1.fasta,Replicase protein,16716, c -> t, S, NA
orf1.fasta,Replicase protein,16749, g -> a, S, NA
orf1.fasta,Replicase protein,16758, c -> t, S, NA
orf1.fasta,Replicase protein,16779, a -> t, S, NA
orf1.fasta,Replicase protein,16794, g -> a, S, NA

orf1.fasta,Replicase protein,16821, c -> t, S, NA
orf1.fasta,Replicase protein,16830, a -> t, S, NA
orf1.fasta,Replicase protein,16860, c -> t, S, NA
orf1.fasta,Replicase protein,16875, t -> c, S, NA
orf1.fasta,Replicase protein,16890, c -> t, S, NA
orf1.fasta,Replicase protein,16905, g -> a, S, NA
orf1.fasta,Replicase protein,16908, t -> g, S, NA
orf1.fasta,Replicase protein,16965, c -> t, S, NA
orf1.fasta,Replicase protein,16983, a -> g, S, NA
orf1.fasta,Replicase protein,17034, c -> t, S, NA
orf1.fasta,Replicase protein,17064, c -> t, S, NA
orf1.fasta,Replicase protein,17082, t -> c, S, NA
orf1.fasta,Replicase protein,17091, c -> t, S, NA
orf1.fasta,Replicase protein,17142, a -> g, S, NA
orf1.fasta,Replicase protein,17151, c -> t, S, NA
orf1.fasta,Replicase protein,17172, c -> t, S, NA
orf1.fasta,Replicase protein,17232, t -> c, S, NA
orf1.fasta,Replicase protein,17247, c -> t, S, NA
orf1.fasta,Replicase protein,17319, g -> a, S, NA
orf1.fasta,Replicase protein,17397, t -> c, S, NA
orf1.fasta,Replicase protein,17400, c -> t, S, NA
orf1.fasta,Replicase protein,17439, t -> c, S, NA
orf1.fasta,Replicase protein,17469, a -> t, S, NA
orf1.fasta,Replicase protein,17472, a -> g, S, NA
orf1.fasta,Replicase protein,17511, c -> t, S, NA
orf1.fasta,Replicase protein,17577, c -> t, S, NA
orf1.fasta,Replicase protein,17587, a -> c, S, NA
orf1.fasta,Replicase protein,17673, g -> t, S, NA
orf1.fasta,Replicase protein,17685, c -> t, S, NA
orf1.fasta,Replicase protein,17693, a -> g, N, K -> R
orf1.fasta,Replicase protein,17709, t -> c, S, NA
orf1.fasta,Replicase protein,17735, a -> g, N, N -> S
orf1.fasta,Replicase protein,17758, t -> c, N, Y -> H
orf1.fasta,Replicase protein,17760, t -> c, N, Y -> H
orf1.fasta,Replicase protein,17883, g -> t, S, NA
orf1.fasta,Replicase protein,17898, c -> t, S, NA
orf1.fasta,Replicase protein,17946, g -> t, S, NA
orf1.fasta,Replicase protein,17976, t -> c, S, NA
orf1.fasta,Replicase protein,18033, c -> t, S, NA
orf1.fasta,Replicase protein,18141, c -> t, S, NA
orf1.fasta,Replicase protein,18243, g -> a, S, NA
orf1.fasta,Replicase protein,18252, g -> a, S, NA
orf1.fasta,Replicase protein,18258, t -> a, S, NA
orf1.fasta,Replicase protein,18276, a -> t, S, NA
orf1.fasta,Replicase protein,18282, c -> t, S, NA
orf1.fasta,Replicase protein,18285, t -> a, S, NA
orf1.fasta,Replicase protein,18293, t -> c, N, V -> A
orf1.fasta,Replicase protein,18300, t -> a, S, NA
orf1.fasta,Replicase protein,18303, a -> t, S, NA
orf1.fasta,Replicase protein,18312, t -> c, S, NA

orf1.fasta,Replicase protein,18314, g -> c, N, C -> S
orf1.fasta,Replicase protein,18344, t -> c, N, V -> A
orf1.fasta,Replicase protein,18348, c -> t, S, NA
orf1.fasta,Replicase protein,18446, c -> t, N, T -> I
orf1.fasta,Replicase protein,18450, t -> a, S, NA
orf1.fasta,Replicase protein,18483, c -> t, S, NA
orf1.fasta,Replicase protein,18484, a -> g, N, I -> V
orf1.fasta,Replicase protein,18510, c -> a, S, NA
orf1.fasta,Replicase protein,18552, c -> t, S, NA
orf1.fasta,Replicase protein,18564, c -> t, S, NA
orf1.fasta,Replicase protein,18591, c -> t, S, NA
orf1.fasta,Replicase protein,18657, c -> a, S, NA
orf1.fasta,Replicase protein,18669, t -> c, S, NA
orf1.fasta,Replicase protein,18672, g -> a, S, NA
orf1.fasta,Replicase protein,18677, g -> c, N, S -> T
orf1.fasta,Replicase protein,18696, t -> c, S, NA
orf1.fasta,Replicase protein,18699, t -> c, S, NA
orf1.fasta,Replicase protein,18702, t -> a, S, NA
orf1.fasta,Replicase protein,18708, g -> t, S, NA
orf1.fasta,Replicase protein,18735, c -> t, S, NA
orf1.fasta,Replicase protein,18747, c -> t, S, NA
orf1.fasta,Replicase protein,18789, a -> t, S, NA
orf1.fasta,Replicase protein,18790, t -> c, N, F -> L
orf1.fasta,Replicase protein,18843, c -> t, S, NA
orf1.fasta,Replicase protein,18864, t -> g, S, NA
orf1.fasta,Replicase protein,18930, g -> a, S, NA
orf1.fasta,Replicase protein,18951, g -> a, S, NA
orf1.fasta,Replicase protein,18966, c -> t, S, NA
orf1.fasta,Replicase protein,18994, t -> c, N, Y -> H
orf1.fasta,Replicase protein,19017, c -> t, S, NA
orf1.fasta,Replicase protein,19038, t -> c, S, NA
orf1.fasta,Replicase protein,19095, c -> a, S, NA
orf1.fasta,Replicase protein,19098, g -> t, S, NA
orf1.fasta,Replicase protein,19104, t -> c, S, NA
orf1.fasta,Replicase protein,19107, t -> a, S, NA
orf1.fasta,Replicase protein,19125, c -> t, S, NA
orf1.fasta,Replicase protein,19167, t -> a, S, NA
orf1.fasta,Replicase protein,19203, c -> t, S, NA
orf1.fasta,Replicase protein,19248, a -> g, S, NA
orf1.fasta,Replicase protein,19254, t -> c, S, NA
orf1.fasta,Replicase protein,19257, g -> a, S, NA
orf1.fasta,Replicase protein,19266, t -> c, S, NA
orf1.fasta,Replicase protein,19275, t -> c, S, NA
orf1.fasta,Replicase protein,19296, g -> a, S, NA
orf1.fasta,Replicase protein,19302, c -> t, S, NA
orf1.fasta,Replicase protein,19311, g -> a, S, NA
orf1.fasta,Replicase protein,19313, a -> g, N, E -> G
orf1.fasta,Replicase protein,19350, c -> t, S, NA
orf1.fasta,Replicase protein,19353, a -> g, S, NA
orf1.fasta,Replicase protein,19356, c -> t, S, NA

orf1.fasta,Replicase protein,19386, t -> c, S, NA
orf1.fasta,Replicase protein,19394, c -> t, N, T -> I
orf1.fasta,Replicase protein,19440, t -> a, S, NA
orf1.fasta,Replicase protein,19443, a -> g, S, NA
orf1.fasta,Replicase protein,19455, g -> a, S, NA
orf1.fasta,Replicase protein,19492, g -> a, N, V -> I
orf1.fasta,Replicase protein,19494, t -> a, N, V -> I
orf1.fasta,Replicase protein,19497, t -> c, S, NA
orf1.fasta,Replicase protein,19557, t -> c, S, NA
orf1.fasta,Replicase protein,19617, c -> t, S, NA
orf1.fasta,Replicase protein,19679, t -> c, N, M -> T
orf1.fasta,Replicase protein,19692, a -> g, S, NA
orf1.fasta,Replicase protein,19707, t -> a, S, NA
orf1.fasta,Replicase protein,19731, g -> a, S, NA
orf1.fasta,Replicase protein,19734, c -> t, S, NA
orf1.fasta,Replicase protein,19740, t -> a, S, NA
orf1.fasta,Replicase protein,19749, t -> c, S, NA
orf1.fasta,Replicase protein,19752, a -> g, S, NA
orf1.fasta,Replicase protein,19797, a -> g, S, NA
orf1.fasta,Replicase protein,19834, g -> a, N, G -> R
orf1.fasta,Replicase protein,19908, a -> t, N, E -> D
orf1.fasta,Replicase protein,19915, g -> a, N, A -> T
orf1.fasta,Replicase protein,19923, t -> c, S, NA
orf1.fasta,Replicase protein,19926, c -> t, S, NA
orf1.fasta,Replicase protein,19968, t -> a, S, NA
orf1.fasta,Replicase protein,19974, c -> t, S, NA
orf1.fasta,Replicase protein,20007, t -> c, S, NA
orf1.fasta,Replicase protein,20037, t -> c, S, NA
orf1.fasta,Replicase protein,20043, t -> c, S, NA
orf1.fasta,Replicase protein,20052, t -> c, S, NA
orf1.fasta,Replicase protein,20070, t -> c, S, NA
orf1.fasta,Replicase protein,20082, a -> t, S, NA
orf1.fasta,Replicase protein,20130, g -> t, S, NA
orf1.fasta,Replicase protein,20134, t -> a, N, Y -> N
orf1.fasta,Replicase protein,20143, g -> a, N, V -> I
orf1.fasta,Replicase protein,20160, g -> t, S, NA
orf1.fasta,Replicase protein,20163, c -> t, S, NA
orf1.fasta,Replicase protein,20184, t -> c, S, NA
orf1.fasta,Replicase protein,20199, a -> g, S, NA
orf1.fasta,Replicase protein,20209, a -> g, N, S -> G
orf1.fasta,Replicase protein,20250, c -> t, S, NA
orf1.fasta,Replicase protein,20268, t -> c, S, NA
orf1.fasta,Replicase protein,20310, t -> a, S, NA
orf1.fasta,Replicase protein,20337, t -> c, S, NA
orf1.fasta,Replicase protein,20364, c -> t, S, NA
orf1.fasta,Replicase protein,20376, c -> t, S, NA
orf1.fasta,Replicase protein,20469, c -> t, S, NA
orf1.fasta,Replicase protein,20577, t -> c, S, NA
orf1.fasta,Replicase protein,20586, c -> a, S, NA
orf1.fasta,Replicase protein,20589, c -> t, S, NA

orf1.fasta,Replicase protein,20772, t -> c, S, NA
orf1.fasta,Replicase protein,20784, t -> c, S, NA
orf1.fasta,Replicase protein,20820, g -> a, S, NA
orf1.fasta,Replicase protein,20844, c -> t, S, NA
orf1.fasta,Replicase protein,20877, t -> g, S, NA
orf1.fasta,Replicase protein,21138, t -> c, S, NA
orf1.fasta,Replicase protein,21147, a -> g, S, NA
orf1.fasta,Replicase protein,21165, g -> a, S, NA
orf1.fasta,Replicase protein,21178, g -> a, N, A -> T
orf1.fasta,Replicase protein,21204, c -> t, S, NA
orf1.fasta,Replicase protein,21232, a -> g, N, I -> V
orf1.fasta,Replicase protein,21234, a -> t, N, I -> V
orf1.fasta,Replicase protein,21264, g -> t, S, NA
orf2.fasta,Non-structural protein 2,6, c -> a, S, NA
orf2.fasta,Non-structural protein 2,10, t -> g, N, S -> A
orf2.fasta,Non-structural protein 2,12, a -> t, N, S -> A
orf2.fasta,Non-structural protein 2,24, t -> g, N, N -> K
orf2.fasta,Non-structural protein 2,27, g -> t, S, NA
orf2.fasta,Non-structural protein 2,33, t -> c, S, NA
orf2.fasta,Non-structural protein 2,61, g -> c, N, D -> Q
orf2.fasta,Non-structural protein 2,63, t -> g, N, D -> Q
orf2.fasta,Non-structural protein 2,68, c -> t, N, S -> F
orf2.fasta,Non-structural protein 2,84, g -> a, S, NA
orf2.fasta,Non-structural protein 2,95, c -> t, N, S -> F
orf2.fasta,Non-structural protein 2,138, a -> g, S, NA
orf2.fasta,Non-structural protein 2,154, a -> g, N, T -> A
orf2.fasta,Non-structural protein 2,156, c -> t, N, T -> A
orf2.fasta,Non-structural protein 2,168, a -> t, S, NA
orf2.fasta,Non-structural protein 2,185, t -> g, N, I -> R
orf2.fasta,Non-structural protein 2,240, t -> c, S, NA
orf2.fasta,Non-structural protein 2,252, g -> a, S, NA
orf2.fasta,Non-structural protein 2,258, t -> a, N, D -> E
orf2.fasta,Non-structural protein 2,261, c -> a, N, N -> K
orf2.fasta,Non-structural protein 2,267, c -> t, S, NA
orf2.fasta,Non-structural protein 2,283, t -> a, N, L -> I
orf2.fasta,Non-structural protein 2,294, c -> t, S, NA
orf2.fasta,Non-structural protein 2,312, g -> a, S, NA
orf2.fasta,Non-structural protein 2,360, c -> t, S, NA
orf2.fasta,Non-structural protein 2,372, a -> g, S, NA
orf2.fasta,Non-structural protein 2,384, c -> a, S, NA
orf2.fasta,Non-structural protein 2,403, t -> c, N, F -> L
orf2.fasta,Non-structural protein 2,429, a -> g, S, NA
orf2.fasta,Non-structural protein 2,450, t -> c, S, NA
orf2.fasta,Non-structural protein 2,459, g -> a, S, NA
orf2.fasta,Non-structural protein 2,465, t -> c, S, NA
orf2.fasta,Non-structural protein 2,477, c -> t, S, NA
orf2.fasta,Non-structural protein 2,486, t -> c, S, NA
orf2.fasta,Non-structural protein 2,495, c -> g, S, NA
orf2.fasta,Non-structural protein 2,513, g -> t, S, NA
orf2.fasta,Non-structural protein 2,534, g -> a, S, NA

orf2.fasta,Non-structural protein 2,553, g -> a, N, V -> I
orf2.fasta,Non-structural protein 2,557, t -> c, N, V -> A
orf2.fasta,Non-structural protein 2,564, t -> c, S, NA
orf2.fasta,Non-structural protein 2,580, c -> a, N, H -> K
orf2.fasta,Non-structural protein 2,582, t -> g, N, H -> K
orf2.fasta,Non-structural protein 2,583, t -> a, N, * -> K
orf3.fasta,hemagglutinin-esterase protein,17, t -> g, N, I -> R
orf3.fasta,hemagglutinin-esterase protein,22, t -> g, N, F -> V
orf3.fasta,hemagglutinin-esterase protein,34, a -> t, N, S -> C
orf3.fasta,hemagglutinin-esterase protein,39, c -> a, S, NA
orf3.fasta,hemagglutinin-esterase protein,45, c -> t, S, NA
orf3.fasta,hemagglutinin-esterase protein,47, c -> g, N, T -> S
orf3.fasta,hemagglutinin-esterase protein,48, t -> c, N, T -> S
orf3.fasta,hemagglutinin-esterase protein,49, t -> c, N, F -> Q
orf3.fasta,hemagglutinin-esterase protein,50, t -> a, N, F -> Q
orf3.fasta,hemagglutinin-esterase protein,51, t -> a, N, F -> Q
orf3.fasta,hemagglutinin-esterase protein,60, a -> t, N, E -> D
orf3.fasta,hemagglutinin-esterase protein,69, g -> t, S, NA
orf3.fasta,hemagglutinin-esterase protein,84, a -> g, S, NA
orf3.fasta,hemagglutinin-esterase protein,90, t -> a, N, F -> L
orf3.fasta,hemagglutinin-esterase protein,95, a -> g, N, D -> G
orf3.fasta,hemagglutinin-esterase protein,96, t -> a, N, D -> G
orf3.fasta,hemagglutinin-esterase protein,106, c -> t, S, NA
orf3.fasta,hemagglutinin-esterase protein,114, g -> t, S, NA
orf3.fasta,hemagglutinin-esterase protein,126, t -> a, S, NA
orf3.fasta,hemagglutinin-esterase protein,129, c -> t, S, NA
orf3.fasta,hemagglutinin-esterase protein,146, a -> c, N, N -> T
orf3.fasta,hemagglutinin-esterase protein,147, c -> t, N, N -> T
orf3.fasta,hemagglutinin-esterase protein,189, t -> c, S, NA
orf3.fasta,hemagglutinin-esterase protein,190, c -> t, S, NA
orf3.fasta,hemagglutinin-esterase protein,210, a -> t, S, NA
orf3.fasta,hemagglutinin-esterase protein,276, t -> c, S, NA
orf3.fasta,hemagglutinin-esterase protein,297, t -> c, S, NA
orf3.fasta,hemagglutinin-esterase protein,315, c -> t, S, NA
orf3.fasta,hemagglutinin-esterase protein,322, t -> c, N, Y -> H
orf3.fasta,hemagglutinin-esterase protein,341, c -> t, N, T -> I
orf3.fasta,hemagglutinin-esterase protein,342, t -> c, N, T -> I
orf3.fasta,hemagglutinin-esterase protein,344, g -> c, N, S -> T
orf3.fasta,hemagglutinin-esterase protein,346, g -> t, N, A -> S
orf3.fasta,hemagglutinin-esterase protein,353, a -> g, N, N -> S
orf3.fasta,hemagglutinin-esterase protein,363, a -> t, S, NA
orf3.fasta,hemagglutinin-esterase protein,384, a -> g, S, NA
orf3.fasta,hemagglutinin-esterase protein,408, g -> t, N, K -> N
orf3.fasta,hemagglutinin-esterase protein,417, t -> g, S, NA
orf3.fasta,hemagglutinin-esterase protein,420, c -> t, S, NA
orf3.fasta,hemagglutinin-esterase protein,426, t -> c, S, NA
orf3.fasta,hemagglutinin-esterase protein,435, a -> t, N, L -> F
orf3.fasta,hemagglutinin-esterase protein,444, g -> a, S, NA
orf3.fasta,hemagglutinin-esterase protein,450, c -> t, S, NA
orf3.fasta,hemagglutinin-esterase protein,456, c -> t, S, NA

orf3.fasta,hemagglutinin-esterase protein,462, g -> a, S, NA
orf3.fasta,hemagglutinin-esterase protein,465, a -> t, S, NA
orf3.fasta,hemagglutinin-esterase protein,466, a -> g, N, T -> A
orf3.fasta,hemagglutinin-esterase protein,468, t -> a, N, T -> A
orf3.fasta,hemagglutinin-esterase protein,474, c -> t, S, NA
orf3.fasta,hemagglutinin-esterase protein,477, c -> a, S, NA
orf3.fasta,hemagglutinin-esterase protein,480, a -> c, S, NA
orf3.fasta,hemagglutinin-esterase protein,481, t -> c, N, F -> L
orf3.fasta,hemagglutinin-esterase protein,489, g -> a, S, NA
orf3.fasta,hemagglutinin-esterase protein,495, c -> t, S, NA
orf3.fasta,hemagglutinin-esterase protein,502, a -> g, N, I -> V
orf3.fasta,hemagglutinin-esterase protein,510, c -> t, S, NA
orf3.fasta,hemagglutinin-esterase protein,513, t -> c, S, NA
orf3.fasta,hemagglutinin-esterase protein,528, c -> t, S, NA
orf3.fasta,hemagglutinin-esterase protein,534, g -> a, S, NA
orf3.fasta,hemagglutinin-esterase protein,541, g -> t, N, V -> F
orf3.fasta,hemagglutinin-esterase protein,543, g -> t, N, V -> F
orf3.fasta,hemagglutinin-esterase protein,544, g -> a, N, G -> R
orf3.fasta,hemagglutinin-esterase protein,546, t -> g, N, G -> R
orf3.fasta,hemagglutinin-esterase protein,562, t -> g, N, S -> V
orf3.fasta,hemagglutinin-esterase protein,563, c -> t, N, S -> V
orf3.fasta,hemagglutinin-esterase protein,570, a -> t, S, NA
orf3.fasta,hemagglutinin-esterase protein,578, c -> a, N, S -> Y
orf3.fasta,hemagglutinin-esterase protein,580, c -> t, S, NA
orf3.fasta,hemagglutinin-esterase protein,582, t -> g, S, NA
orf3.fasta,hemagglutinin-esterase protein,624, c -> t, S, NA
orf3.fasta,hemagglutinin-esterase protein,673, t -> c, N, F -> L
orf3.fasta,hemagglutinin-esterase protein,711, t -> c, S, NA
orf3.fasta,hemagglutinin-esterase protein,754, c -> t, S, NA
orf3.fasta,hemagglutinin-esterase protein,756, t -> a, S, NA
orf3.fasta,hemagglutinin-esterase protein,759, a -> t, S, NA
orf3.fasta,hemagglutinin-esterase protein,778, c -> t, S, NA
orf3.fasta,hemagglutinin-esterase protein,822, t -> a, S, NA
orf3.fasta,hemagglutinin-esterase protein,845, c -> a, N, A -> D
orf3.fasta,hemagglutinin-esterase protein,870, t -> c, S, NA
orf3.fasta,hemagglutinin-esterase protein,873, g -> c, S, NA
orf3.fasta,hemagglutinin-esterase protein,918, a -> t, S, NA
orf3.fasta,hemagglutinin-esterase protein,926, t -> c, N, L -> P
orf3.fasta,hemagglutinin-esterase protein,933, c -> t, S, NA
orf3.fasta,hemagglutinin-esterase protein,945, c -> t, S, NA
orf3.fasta,hemagglutinin-esterase protein,955, t -> a, N, S -> T
orf3.fasta,hemagglutinin-esterase protein,960, c -> t, S, NA
orf3.fasta,hemagglutinin-esterase protein,963, c -> t, S, NA
orf3.fasta,hemagglutinin-esterase protein,970, a -> g, N, I -> V
orf3.fasta,hemagglutinin-esterase protein,979, g -> a, N, V -> I
orf3.fasta,hemagglutinin-esterase protein,981, t -> c, N, V -> I
orf3.fasta,hemagglutinin-esterase protein,984, c -> t, S, NA
orf3.fasta,hemagglutinin-esterase protein,990, t -> g, S, NA
orf3.fasta,hemagglutinin-esterase protein,993, t -> c, S, NA
orf3.fasta,hemagglutinin-esterase protein,1026, g -> a, S, NA

orf3.fasta,hemagglutinin-esterase protein,1032, t -> c, S, NA
orf3.fasta,hemagglutinin-esterase protein,1050, g -> a, S, NA
orf3.fasta,hemagglutinin-esterase protein,1057, a -> g, N, I -> V
orf3.fasta,hemagglutinin-esterase protein,1077, c -> t, S, NA
orf3.fasta,hemagglutinin-esterase protein,1082, c -> g, N, T -> S
orf3.fasta,hemagglutinin-esterase protein,1086, t -> c, S, NA
orf3.fasta,hemagglutinin-esterase protein,1095, t -> c, S, NA
orf3.fasta,hemagglutinin-esterase protein,1099, c -> t, N, P -> X
orf3.fasta,hemagglutinin-esterase protein,1101, t -> y, N, P -> X
orf3.fasta,hemagglutinin-esterase protein,1107, t -> c, S, NA
orf3.fasta,hemagglutinin-esterase protein,1109, a -> g, N, N -> R
orf3.fasta,hemagglutinin-esterase protein,1110, t -> a, N, N -> R
orf3.fasta,hemagglutinin-esterase protein,1126, a -> g, N, S -> D
orf3.fasta,hemagglutinin-esterase protein,1127, g -> a, N, S -> D
orf3.fasta,hemagglutinin-esterase protein,1133, t -> a, N, I -> N
orf3.fasta,hemagglutinin-esterase protein,1135, t -> a, N, F -> T
orf3.fasta,hemagglutinin-esterase protein,1136, t -> c, N, F -> T
orf3.fasta,hemagglutinin-esterase protein,1138, t -> c, N, F -> P
orf3.fasta,hemagglutinin-esterase protein,1139, t -> c, N, F -> P
orf3.fasta,hemagglutinin-esterase protein,1144, t -> g, N, L -> V
orf3.fasta,hemagglutinin-esterase protein,1149, t -> c, S, NA
orf3.fasta,hemagglutinin-esterase protein,1152, a -> t, S, NA
orf3.fasta,hemagglutinin-esterase protein,1158, t -> g, S, NA
orf3.fasta,hemagglutinin-esterase protein,1164, c -> t, S, NA
orf3.fasta,hemagglutinin-esterase protein,1167, a -> g, S, NA
orf3.fasta,hemagglutinin-esterase protein,1180, c -> t, S, NA
orf3.fasta,hemagglutinin-esterase protein,1197, a -> g, S, NA
orf3.fasta,hemagglutinin-esterase protein,1203, c -> t, S, NA
orf3.fasta,hemagglutinin-esterase protein,1213, g -> a, N, V -> I
orf3.fasta,hemagglutinin-esterase protein,1218, a -> t, S, NA
orf3.fasta,hemagglutinin-esterase protein,1220, g -> t, N, G -> V
orf3.fasta,hemagglutinin-esterase protein,1221, t -> a, N, G -> V
orf3.fasta,hemagglutinin-esterase protein,1226, c -> t, N, S -> L
orf3.fasta,hemagglutinin-esterase protein,1231, g -> t, N, V -> L
orf3.fasta,hemagglutinin-esterase protein,1267, t -> g, N, Y -> D
orf3.fasta,hemagglutinin-esterase protein,1275, a -> g, S, NA
orf4.fasta,spike glycoprotein,9, t -> g, N, F -> L
orf4.fasta,spike glycoprotein,21, c -> t, S, NA
orf4.fasta,spike glycoprotein,25, c -> t, S, NA
orf4.fasta,spike glycoprotein,27, g -> a, S, NA
orf4.fasta,spike glycoprotein,30, t -> a, S, NA
orf4.fasta,spike glycoprotein,31, t -> a, N, S -> T
orf4.fasta,spike glycoprotein,33, t -> g, N, S -> T
orf4.fasta,spike glycoprotein,42, a -> t, S, NA
orf4.fasta,spike glycoprotein,46, t -> a, N, L -> I
orf4.fasta,spike glycoprotein,51, g -> c, S, NA
orf4.fasta,spike glycoprotein,65, a -> c, N, N -> T
orf4.fasta,spike glycoprotein,69, t -> a, S, NA
orf4.fasta,spike glycoprotein,70, t -> g, N, S -> V
orf4.fasta,spike glycoprotein,71, c -> t, N, S -> V

orf4.fasta,spike glycoprotein,72, a -> t, N, S -> V
orf4.fasta,spike glycoprotein,75, a -> c, S, NA
orf4.fasta,spike glycoprotein,84, c -> t, S, NA
orf4.fasta,spike glycoprotein,99, g -> t, S, NA
orf4.fasta,spike glycoprotein,102, a -> t, S, NA
orf4.fasta,spike glycoprotein,112, t -> a, N, S -> T
orf4.fasta,spike glycoprotein,117, a -> t, N, E -> D
orf4.fasta,spike glycoprotein,118, g -> a, N, V -> T
orf4.fasta,spike glycoprotein,119, t -> c, N, V -> T
orf4.fasta,spike glycoprotein,123, t -> c, S, NA
orf4.fasta,spike glycoprotein,129, c -> t, S, NA
orf4.fasta,spike glycoprotein,141, g -> a, S, NA
orf4.fasta,spike glycoprotein,144, g -> t, S, NA
orf4.fasta,spike glycoprotein,149, t -> a, N, F -> Y
orf4.fasta,spike glycoprotein,168, c -> g, S, NA
orf4.fasta,spike glycoprotein,180, c -> t, S, NA
orf4.fasta,spike glycoprotein,183, a -> c, S, NA
orf4.fasta,spike glycoprotein,192, c -> t, S, NA
orf4.fasta,spike glycoprotein,201, t -> c, S, NA
orf4.fasta,spike glycoprotein,204, c -> t, S, NA
orf4.fasta,spike glycoprotein,207, a -> t, S, NA
orf4.fasta,spike glycoprotein,209, t -> c, N, I -> T
orf4.fasta,spike glycoprotein,217, g -> t, N, A -> S
orf4.fasta,spike glycoprotein,224, t -> a, N, F -> Y
orf4.fasta,spike glycoprotein,237, t -> a, S, NA
orf4.fasta,spike glycoprotein,243, a -> g, S, NA
orf4.fasta,spike glycoprotein,250, c -> t, N, R -> L
orf4.fasta,spike glycoprotein,251, g -> t, N, R -> L
orf4.fasta,spike glycoprotein,253, t -> c, S, NA
orf4.fasta,spike glycoprotein,264, c -> a, S, NA
orf4.fasta,spike glycoprotein,265, t -> c, S, NA
orf4.fasta,spike glycoprotein,267, g -> a, S, NA
orf4.fasta,spike glycoprotein,276, g -> a, S, NA
orf4.fasta,spike glycoprotein,279, g -> a, S, NA
orf4.fasta,spike glycoprotein,286, t -> c, S, NA
orf4.fasta,spike glycoprotein,288, a -> t, S, NA
orf4.fasta,spike glycoprotein,291, a -> t, S, NA
orf4.fasta,spike glycoprotein,292, c -> g, N, P -> D
orf4.fasta,spike glycoprotein,293, c -> a, N, P -> D
orf4.fasta,spike glycoprotein,294, g -> t, N, P -> D
orf4.fasta,spike glycoprotein,299, a -> c, N, N -> T
orf4.fasta,spike glycoprotein,301, g -> a, N, D -> N
orf4.fasta,spike glycoprotein,321, t -> c, S, NA
orf4.fasta,spike glycoprotein,327, c -> t, S, NA
orf4.fasta,spike glycoprotein,329, g -> c, N, S -> T
orf4.fasta,spike glycoprotein,332, g -> a, N, R -> K
orf4.fasta,spike glycoprotein,333, a -> g, N, R -> K
orf4.fasta,spike glycoprotein,334, t -> g, N, F -> V
orf4.fasta,spike glycoprotein,337, t -> a, N, F -> I
orf4.fasta,spike glycoprotein,339, c -> t, N, F -> I

orf4.fasta,spike glycoprotein,351, t -> a, S, NA
orf4.fasta,spike glycoprotein,353, t -> a, N, I -> K
orf4.fasta,spike glycoprotein,354, t -> g, N, I -> K
orf4.fasta,spike glycoprotein,375, t -> a, S, NA
orf4.fasta,spike glycoprotein,402, t -> a, S, NA
orf4.fasta,spike glycoprotein,411, c -> t, S, NA
orf4.fasta,spike glycoprotein,412, a -> g, N, I -> V
orf4.fasta,spike glycoprotein,414, a -> g, N, I -> V
orf4.fasta,spike glycoprotein,421, g -> c, N, E -> L
orf4.fasta,spike glycoprotein,422, a -> t, N, E -> L
orf4.fasta,spike glycoprotein,423, g -> a, N, E -> L
orf4.fasta,spike glycoprotein,426, t -> a, S, NA
orf4.fasta,spike glycoprotein,433, t -> a, N, L -> T
orf4.fasta,spike glycoprotein,434, t -> c, N, L -> T
orf4.fasta,spike glycoprotein,435, a -> c, N, L -> T
orf4.fasta,spike glycoprotein,436, c -> a, N, L -> N
orf4.fasta,spike glycoprotein,437, t -> a, N, L -> N
orf4.fasta,spike glycoprotein,439, a -> t, N, I -> L
orf4.fasta,spike glycoprotein,441, t -> g, N, I -> L
orf4.fasta,spike glycoprotein,442, a -> g, N, N -> D
orf4.fasta,spike glycoprotein,445, g -> a, N, G -> N
orf4.fasta,spike glycoprotein,446, g -> a, N, G -> N
orf4.fasta,spike glycoprotein,450, t -> a, N, N -> K
orf4.fasta,spike glycoprotein,462, g -> c, N, L -> F
orf4.fasta,spike glycoprotein,465, g -> a, S, NA
orf4.fasta,spike glycoprotein,466, c -> g, N, Q -> E
orf4.fasta,spike glycoprotein,468, a -> g, N, Q -> E
orf4.fasta,spike glycoprotein,480, t -> c, S, NA
orf4.fasta,spike glycoprotein,483, a -> g, S, NA
orf4.fasta,spike glycoprotein,486, c -> t, S, NA
orf4.fasta,spike glycoprotein,495, t -> c, S, NA
orf4.fasta,spike glycoprotein,498, a -> g, S, NA
orf4.fasta,spike glycoprotein,505, c -> a, N, H -> N
orf4.fasta,spike glycoprotein,510, t -> g, S, NA
orf4.fasta,spike glycoprotein,517, c -> a, N, H -> N
orf4.fasta,spike glycoprotein,526, t -> c, S, NA
orf4.fasta,spike glycoprotein,541, a -> g, N, I -> V
orf4.fasta,spike glycoprotein,557, a -> g, N, Y -> W
orf4.fasta,spike glycoprotein,558, t -> g, N, Y -> W
orf4.fasta,spike glycoprotein,561, c -> t, S, NA
orf4.fasta,spike glycoprotein,566, a -> g, N, D -> G
orf4.fasta,spike glycoprotein,576, t -> c, S, NA
orf4.fasta,spike glycoprotein,585, c -> t, S, NA
orf4.fasta,spike glycoprotein,587, g -> a, N, R -> K
orf4.fasta,spike glycoprotein,624, a -> g, S, NA
orf4.fasta,spike glycoprotein,630, t -> c, S, NA
orf4.fasta,spike glycoprotein,642, g -> a, S, NA
orf4.fasta,spike glycoprotein,651, c -> t, S, NA
orf4.fasta,spike glycoprotein,654, g -> t, S, NA
orf4.fasta,spike glycoprotein,666, c -> t, S, NA

orf4.fasta,spike glycoprotein,675, t -> c, S, NA
orf4.fasta,spike glycoprotein,682, t -> g, N, F -> V
orf4.fasta,spike glycoprotein,687, g -> c, S, NA
orf4.fasta,spike glycoprotein,690, c -> t, S, NA
orf4.fasta,spike glycoprotein,705, g -> t, N, K -> N
orf4.fasta,spike glycoprotein,706, t -> g, N, L -> V
orf4.fasta,spike glycoprotein,708, g -> t, N, L -> V
orf4.fasta,spike glycoprotein,720, t -> g, S, NA
orf4.fasta,spike glycoprotein,726, g -> t, S, NA
orf4.fasta,spike glycoprotein,741, t -> c, S, NA
orf4.fasta,spike glycoprotein,747, a -> t, S, NA
orf4.fasta,spike glycoprotein,757, g -> a, N, D -> N
orf4.fasta,spike glycoprotein,762, c -> t, S, NA
orf4.fasta,spike glycoprotein,765, t -> c, S, NA
orf4.fasta,spike glycoprotein,766, t -> c, S, NA
orf4.fasta,spike glycoprotein,768, a -> g, S, NA
orf4.fasta,spike glycoprotein,769, t -> a, N, S -> T
orf4.fasta,spike glycoprotein,799, a -> t, N, T -> S
orf4.fasta,spike glycoprotein,803, g -> a, N, R -> K
orf4.fasta,spike glycoprotein,809, t -> a, N, F -> Y
orf4.fasta,spike glycoprotein,813, t -> a, S, NA
orf4.fasta,spike glycoprotein,819, c -> t, S, NA
orf4.fasta,spike glycoprotein,822, t -> c, S, NA
orf4.fasta,spike glycoprotein,823, g -> a, N, D -> N
orf4.fasta,spike glycoprotein,825, c -> t, N, D -> N
orf4.fasta,spike glycoprotein,828, g -> a, S, NA
orf4.fasta,spike glycoprotein,838, t -> a, N, L -> I
orf4.fasta,spike glycoprotein,840, a -> t, N, L -> I
orf4.fasta,spike glycoprotein,842, a -> t, N, Y -> F
orf4.fasta,spike glycoprotein,843, c -> t, N, Y -> F
orf4.fasta,spike glycoprotein,844, c -> a, N, H -> N
orf4.fasta,spike glycoprotein,859, g -> a, N, A -> K
orf4.fasta,spike glycoprotein,860, c -> a, N, A -> K
orf4.fasta,spike glycoprotein,861, t -> g, N, A -> K
orf4.fasta,spike glycoprotein,884, t -> a, N, M -> K
orf4.fasta,spike glycoprotein,894, t -> a, S, NA
orf4.fasta,spike glycoprotein,895, t -> c, N, S -> L
orf4.fasta,spike glycoprotein,896, c -> t, N, S -> L
orf4.fasta,spike glycoprotein,897, t -> a, N, S -> L
orf4.fasta,spike glycoprotein,900, a -> t, S, NA
orf4.fasta,spike glycoprotein,903, t -> a, S, NA
orf4.fasta,spike glycoprotein,904, a -> g, N, T -> A
orf4.fasta,spike glycoprotein,910, c -> t, N, P -> S
orf4.fasta,spike glycoprotein,928, c -> t, S, NA
orf4.fasta,spike glycoprotein,1872, c -> t, S, NA
orf4.fasta,spike glycoprotein,1878, a -> t, S, NA
orf4.fasta,spike glycoprotein,1881, c -> t, S, NA
orf4.fasta,spike glycoprotein,1884, g -> a, S, NA
orf4.fasta,spike glycoprotein,1887, c -> t, S, NA
orf4.fasta,spike glycoprotein,1890, a -> t, S, NA

orf4.fasta,spike glycoprotein,1891, c -> t, N, L -> F
orf4.fasta,spike glycoprotein,1894, a -> g, N, I -> V
orf4.fasta,spike glycoprotein,1896, a -> t, N, I -> V
orf4.fasta,spike glycoprotein,1899, a -> g, S, NA
orf4.fasta,spike glycoprotein,1908, c -> g, S, NA
orf4.fasta,spike glycoprotein,1932, t -> c, S, NA
orf4.fasta,spike glycoprotein,1936, c -> t, S, NA
orf4.fasta,spike glycoprotein,1938, t -> a, S, NA
orf4.fasta,spike glycoprotein,1949, g -> a, N, S -> N
orf4.fasta,spike glycoprotein,2394, a -> g, S, NA
orf4.fasta,spike glycoprotein,2400, c -> t, S, NA
orf4.fasta,spike glycoprotein,2409, g -> a, S, NA
orf4.fasta,spike glycoprotein,2415, c -> t, S, NA
orf4.fasta,spike glycoprotein,2421, g -> a, S, NA
orf4.fasta,spike glycoprotein,2430, a -> t, S, NA
orf4.fasta,spike glycoprotein,2433, c -> t, S, NA
orf4.fasta,spike glycoprotein,2437, g -> t, N, A -> S
orf4.fasta,spike glycoprotein,2440, a -> g, N, T -> A
orf4.fasta,spike glycoprotein,2442, a -> t, N, T -> A
orf4.fasta,spike glycoprotein,2457, c -> t, S, NA
orf4.fasta,spike glycoprotein,2469, t -> c, S, NA
orf4.fasta,spike glycoprotein,2471, g -> a, N, R -> K
orf4.fasta,spike glycoprotein,2473, c -> t, N, Q -> S
orf4.fasta,spike glycoprotein,2474, a -> c, N, Q -> S
orf4.fasta,spike glycoprotein,2481, a -> g, S, NA
orf4.fasta,spike glycoprotein,2483, c -> t, N, A -> V
orf4.fasta,spike glycoprotein,2484, c -> t, N, A -> V
orf4.fasta,spike glycoprotein,2487, g -> a, S, NA
orf4.fasta,spike glycoprotein,2496, t -> c, S, NA
orf4.fasta,spike glycoprotein,2499, t -> c, S, NA
orf4.fasta,spike glycoprotein,2505, g -> c, N, E -> D
orf4.fasta,spike glycoprotein,2508, c -> t, S, NA
orf4.fasta,spike glycoprotein,2515, g -> t, N, A -> S
orf4.fasta,spike glycoprotein,2525, t -> c, N, I -> T
orf4.fasta,spike glycoprotein,2539, c -> t, S, NA
orf4.fasta,spike glycoprotein,2548, a -> g, N, T -> A
orf4.fasta,spike glycoprotein,2592, c -> t, S, NA
orf4.fasta,spike glycoprotein,2598, t -> c, S, NA
orf4.fasta,spike glycoprotein,2605, a -> c, N, I -> L
orf4.fasta,spike glycoprotein,2610, g -> a, S, NA
orf4.fasta,spike glycoprotein,2617, a -> t, N, I -> F
orf4.fasta,spike glycoprotein,2631, t -> a, S, NA
orf4.fasta,spike glycoprotein,2637, t -> c, S, NA
orf4.fasta,spike glycoprotein,2643, c -> t, S, NA
orf4.fasta,spike glycoprotein,2646, c -> t, S, NA
orf4.fasta,spike glycoprotein,2676, a -> t, N, E -> D
orf4.fasta,spike glycoprotein,2679, t -> c, S, NA
orf4.fasta,spike glycoprotein,2684, g -> a, N, R -> K
orf4.fasta,spike glycoprotein,2687, c -> t, N, A -> V
orf4.fasta,spike glycoprotein,2693, c -> g, N, T -> S

orf4.fasta,spike glycoprotein,2722, g -> t, N, D -> S
orf4.fasta,spike glycoprotein,2723, a -> c, N, D -> S
orf4.fasta,spike glycoprotein,2724, t -> a, N, D -> S
orf4.fasta,spike glycoprotein,2733, a -> g, S, NA
orf4.fasta,spike glycoprotein,2736, g -> a, S, NA
orf4.fasta,spike glycoprotein,2748, c -> t, S, NA
orf4.fasta,spike glycoprotein,2754, a -> t, S, NA
orf4.fasta,spike glycoprotein,2755, c -> g, N, Q -> E
orf4.fasta,spike glycoprotein,2760, c -> t, S, NA
orf4.fasta,spike glycoprotein,2769, c -> t, S, NA
orf4.fasta,spike glycoprotein,2772, c -> t, S, NA
orf4.fasta,spike glycoprotein,2796, t -> c, S, NA
orf4.fasta,spike glycoprotein,2823, t -> c, S, NA
orf4.fasta,spike glycoprotein,2829, a -> g, S, NA
orf4.fasta,spike glycoprotein,2842, t -> c, S, NA
orf4.fasta,spike glycoprotein,2845, t -> c, S, NA
orf4.fasta,spike glycoprotein,2847, a -> c, S, NA
orf4.fasta,spike glycoprotein,2850, t -> a, S, NA
orf4.fasta,spike glycoprotein,2868, t -> a, S, NA
orf4.fasta,spike glycoprotein,2880, a -> t, S, NA
orf4.fasta,spike glycoprotein,2887, g -> t, N, A -> S
orf4.fasta,spike glycoprotein,2896, t -> c, S, NA
orf4.fasta,spike glycoprotein,2898, a -> g, S, NA
orf4.fasta,spike glycoprotein,2901, c -> t, S, NA
orf4.fasta,spike glycoprotein,2904, t -> c, S, NA
orf4.fasta,spike glycoprotein,2911, a -> t, N, T -> S
orf4.fasta,spike glycoprotein,2916, t -> a, S, NA
orf4.fasta,spike glycoprotein,2934, t -> c, S, NA
orf4.fasta,spike glycoprotein,2964, g -> t, S, NA
orf4.fasta,spike glycoprotein,2965, c -> a, N, L -> I
orf4.fasta,spike glycoprotein,2970, c -> t, S, NA
orf4.fasta,spike glycoprotein,2973, c -> t, S, NA
orf4.fasta,spike glycoprotein,2976, t -> c, S, NA
orf4.fasta,spike glycoprotein,2991, c -> t, S, NA
orf4.fasta,spike glycoprotein,3014, g -> a, N, S -> N
orf4.fasta,spike glycoprotein,3035, a -> g, N, D -> G
orf4.fasta,spike glycoprotein,3054, c -> t, S, NA
orf4.fasta,spike glycoprotein,3057, c -> t, S, NA
orf4.fasta,spike glycoprotein,3060, a -> t, S, NA
orf4.fasta,spike glycoprotein,3087, g -> a, S, NA
orf4.fasta,spike glycoprotein,3088, t -> g, N, S -> A
orf4.fasta,spike glycoprotein,3100, g -> t, N, A -> S
orf4.fasta,spike glycoprotein,3114, a -> t, S, NA
orf4.fasta,spike glycoprotein,3132, g -> a, S, NA
orf4.fasta,spike glycoprotein,3156, c -> t, S, NA
orf4.fasta,spike glycoprotein,3163, g -> t, N, A -> S
orf4.fasta,spike glycoprotein,3165, g -> t, N, A -> S
orf4.fasta,spike glycoprotein,3189, g -> a, S, NA
orf4.fasta,spike glycoprotein,3192, c -> g, S, NA
orf4.fasta,spike glycoprotein,3207, t -> g, S, NA

orf4.fasta,spike glycoprotein,3208, a -> c, N, K -> Q
orf4.fasta,spike glycoprotein,3258, c -> t, S, NA
orf4.fasta,spike glycoprotein,3270, g -> a, S, NA
orf4.fasta,spike glycoprotein,3282, t -> c, S, NA
orf4.fasta,spike glycoprotein,3297, a -> g, S, NA
orf4.fasta,spike glycoprotein,3324, a -> g, S, NA
orf4.fasta,spike glycoprotein,3345, c -> t, S, NA
orf4.fasta,spike glycoprotein,3354, c -> t, S, NA
orf4.fasta,spike glycoprotein,3366, c -> t, S, NA
orf4.fasta,spike glycoprotein,3399, a -> g, S, NA
orf4.fasta,spike glycoprotein,3408, t -> c, S, NA
orf4.fasta,spike glycoprotein,3447, c -> t, S, NA
orf4.fasta,spike glycoprotein,3450, c -> t, S, NA
orf4.fasta,spike glycoprotein,3459, t -> c, S, NA
orf4.fasta,spike glycoprotein,3462, a -> t, S, NA
orf4.fasta,spike glycoprotein,3465, a -> g, S, NA
orf4.fasta,spike glycoprotein,3477, t -> c, S, NA
orf4.fasta,spike glycoprotein,3480, t -> c, S, NA
orf4.fasta,spike glycoprotein,3481, t -> c, S, NA
orf4.fasta,spike glycoprotein,3486, t -> c, S, NA
orf4.fasta,spike glycoprotein,3495, c -> t, S, NA
orf4.fasta,spike glycoprotein,3500, t -> g, N, I -> R
orf4.fasta,spike glycoprotein,3504, a -> t, S, NA
orf4.fasta,spike glycoprotein,3508, t -> g, N, S -> A
orf4.fasta,spike glycoprotein,3510, g -> c, N, S -> A
orf4.fasta,spike glycoprotein,3529, a -> g, N, I -> V
orf4.fasta,spike glycoprotein,3544, t -> a, N, S -> T
orf4.fasta,spike glycoprotein,3591, c -> t, S, NA
orf4.fasta,spike glycoprotein,3592, c -> g, N, Q -> G
orf4.fasta,spike glycoprotein,3593, a -> g, N, Q -> G
orf4.fasta,spike glycoprotein,3603, t -> c, S, NA
orf4.fasta,spike glycoprotein,3607, a -> g, N, M -> V
orf4.fasta,spike glycoprotein,3609, g -> t, N, M -> V
orf4.fasta,spike glycoprotein,3649, c -> g, N, L -> V
orf4.fasta,spike glycoprotein,3662, c -> t, N, T -> I
orf4.fasta,spike glycoprotein,3663, a -> t, N, T -> I
orf4.fasta,spike glycoprotein,3666, g -> a, S, NA
orf4.fasta,spike glycoprotein,3672, t -> c, S, NA
orf4.fasta,spike glycoprotein,3678, t -> c, S, NA
orf4.fasta,spike glycoprotein,3684, c -> t, S, NA
orf4.fasta,spike glycoprotein,3687, c -> t, S, NA
orf4.fasta,spike glycoprotein,3696, a -> g, S, NA
orf4.fasta,spike glycoprotein,3700, t -> g, N, Y -> D
orf4.fasta,spike glycoprotein,3721, t -> a, N, S -> T
orf4.fasta,spike glycoprotein,3723, t -> a, N, S -> T
orf4.fasta,spike glycoprotein,3765, g -> a, S, NA
orf4.fasta,spike glycoprotein,3801, a -> g, S, NA
orf4.fasta,spike glycoprotein,3807, t -> a, S, NA
orf4.fasta,spike glycoprotein,3816, c -> t, S, NA
orf4.fasta,spike glycoprotein,3825, t -> g, N, H -> Q

orf4.fasta,spike glycoprotein,3855, a -> c, S, NA
orf4.fasta,spike glycoprotein,3870, g -> a, S, NA
orf4.fasta,spike glycoprotein,3904, c -> t, N, L -> F
orf4.fasta,spike glycoprotein,3987, c -> t, S, NA
orf4.fasta,spike glycoprotein,4014, c -> t, S, NA
orf4.fasta,spike glycoprotein,4023, t -> a, N, F -> L
orf4.fasta,spike glycoprotein,4029, c -> t, S, NA
orf4.fasta,spike glycoprotein,4035, t -> a, S, NA
orf4.fasta,spike glycoprotein,4044, c -> a, N, D -> E
orf4.fasta,9spike glycoprotein,4047, c -> t, S, NA
orf5.fasta,non-structural protein 9.4,5, c -> a, N, T -> K
orf5.fasta,non-structural protein 9.4,8, c -> t, N, T -> I
orf5.fasta,non-structural protein 9.4,18, c -> t, S, NA
orf5.fasta,non-structural protein 9.4,20, t -> c, N, F -> S
orf5.fasta,non-structural protein 9.4,24, t -> c, S, NA
orf5.fasta,non-structural protein 9.4,28, c -> t, S, NA
orf5.fasta,non-structural protein 9.4,31, a -> m, N, T -> X
orf5.fasta,non-structural protein 9.4,32, c -> y, N, T -> X
orf5.fasta,non-structural protein 9.4,34, c -> y, N, L -> X
orf5.fasta,non-structural protein 9.4,35, t -> m, N, L -> X
orf5.fasta,non-structural protein 9.4,36, t -> k, N, L -> X
orf5.fasta,non-structural protein 9.4,38, a -> w, N, D -> X
orf5.fasta,non-structural protein 9.4,39, c -> m, N, D -> X
orf5.fasta,non-structural protein 9.4,40, g -> a, N, D -> X
orf5.fasta,non-structural protein 9.4,42, t -> y, N, D -> X
orf5.fasta,non-structural protein 9.4,43, a -> m, N, I -> X
orf5.fasta,non-structural protein 9.4,45, a -> w, N, I -> X
orf5.fasta,non-structural protein 9.4,46, g -> w, N, V -> X
orf5.fasta,non-structural protein 9.4,59, t -> c, N, I -> T
orf5.fasta,non-structural protein 9.4,73, t -> a, N, * -> K
orf5.fasta,non-structural protein 9.4,75, a -> g, N, * -> K
orf6.fasta,non-structural protein 12.7,13, t -> a, N, C -> K
orf6.fasta,non-structural protein 12.7,14, g -> a, N, C -> K
orf6.fasta,non-structural protein 12.7,15, c -> a, N, C -> K
orf6.fasta,non-structural protein 12.7,21, a -> g, S, NA
orf6.fasta,non-structural protein 12.7,23, a -> t, N, K -> I
orf6.fasta,non-structural protein 12.7,24, g -> t, N, K -> I
orf6.fasta,non-structural protein 12.7,42, t -> c, S, NA
orf6.fasta,non-structural protein 12.7,48, a -> w, N, G -> X
orf6.fasta,non-structural protein 12.7,54, c -> t, S, NA
orf6.fasta,non-structural protein 12.7,58, t -> c, N, S -> P
orf6.fasta,non-structural protein 12.7,62, t -> a, N, V -> E
orf6.fasta,non-structural protein 12.7,64, c -> t, S, NA
orf6.fasta,non-structural protein 12.7,75, c -> t, S, NA
orf6.fasta,non-structural protein 12.7,86, a -> t, N, Y -> F
orf6.fasta,non-structural protein 12.7,94, c -> a, N, Q -> K
orf6.fasta,non-structural protein 12.7,128, a -> g, N, N -> S
orf6.fasta,non-structural protein 12.7,130, t -> c, N, Y -> H
orf6.fasta,non-structural protein 12.7,147, c -> t, S, NA
orf6.fasta,non-structural protein 12.7,156, t -> c, S, NA

orf6.fasta,non-structural protein 12.7,169, t -> c, N, F -> L
orf6.fasta,non-structural protein 12.7,171, t -> c, N, F -> L
orf6.fasta,non-structural protein 12.7,175, t -> c, N, L -> X
orf6.fasta,non-structural protein 12.7,180, c -> t, S, NA
orf6.fasta,non-structural protein 12.7,197, a -> g, N, Y -> C
orf6.fasta,non-structural protein 12.7,214, a -> g, N, I -> V
orf6.fasta,non-structural protein 12.7,238, t -> g, N, S -> A
orf6.fasta,non-structural protein 12.7,244, t -> a, N, S -> T
orf6.fasta,non-structural protein 12.7,247, g -> t, N, V -> L
orf6.fasta,non-structural protein 12.7,252, a -> g, S, NA
orf6.fasta,non-structural protein 12.7,255, a -> g, S, NA
orf6.fasta,non-structural protein 12.7,261, t -> c, S, NA
orf6.fasta,non-structural protein 12.7,264, t -> c, S, NA
orf6.fasta,non-structural protein 12.7,276, t -> c, S, NA
orf6.fasta,non-structural protein 12.7,291, c -> t, S, NA
orf6.fasta,non-structural protein 12.7,303, a -> g, S, NA
orf6.fasta,non-structural protein 12.7,306, c -> a, N, S -> R
orf6.fasta,non-structural protein 12.7,310, a -> g, N, S -> E
orf6.fasta,non-structural protein 12.7,311, g -> a, N, S -> E
orf6.fasta,non-structural protein 12.7,312, t -> g, N, S -> E
orf7.fasta,envelope protein,93, c -> t, S, NA
orf7.fasta,envelope protein,95, t -> c, N, V -> A
orf7.fasta,envelope protein,96, t -> a, N, V -> A
orf7.fasta,envelope protein,115, t -> a, N, F -> I
orf7.fasta,envelope protein,153, t -> c, S, NA
orf7.fasta,envelope protein,219, g -> t, N, E -> D
orf7.fasta,envelope protein,234, c -> t, S, NA
orf8.fasta,membrane protein,10, c -> t, N, P -> L
orf8.fasta,membrane protein,11, c -> t, N, P -> L
orf8.fasta,membrane protein,23, t -> c, N, V -> A
orf8.fasta,membrane protein,31, a -> t, N, I -> Y
orf8.fasta,membrane protein,32, t -> a, N, I -> Y
orf8.fasta,membrane protein,35, g -> c, N, S -> T
orf8.fasta,membrane protein,78, t -> c, S, NA
orf8.fasta,membrane protein,93, a -> t, S, NA
orf8.fasta,membrane protein,97, g -> c, N, V -> L
orf8.fasta,membrane protein,102, c -> t, S, NA
orf8.fasta,membrane protein,108, c -> t, S, NA
orf8.fasta,membrane protein,112, a -> g, N, I -> V
orf8.fasta,membrane protein,118, c -> t, S, NA
orf8.fasta,membrane protein,120, t -> g, S, NA
orf8.fasta,membrane protein,132, c -> t, S, NA
orf8.fasta,membrane protein,169, g -> a, N, V -> I
orf8.fasta,membrane protein,171, t -> c, N, V -> I
orf8.fasta,membrane protein,175, c -> t, S, NA
orf8.fasta,membrane protein,183, c -> t, S, NA
orf8.fasta,membrane protein,192, t -> c, S, NA
orf8.fasta,membrane protein,195, a -> t, S, NA
orf8.fasta,membrane protein,201, a -> c, S, NA
orf8.fasta,membrane protein,204, t -> c, S, NA

orf8.fasta,membrane protein,213, c -> t, S, NA
orf8.fasta,membrane protein,219, c -> t, S, NA
orf8.fasta,membrane protein,225, a -> g, S, NA
orf8.fasta,membrane protein,228, c -> t, S, NA
orf8.fasta,membrane protein,246, c -> t, S, NA
orf8.fasta,membrane protein,255, c -> t, S, NA
orf8.fasta,membrane protein,279, t -> c, S, NA
orf8.fasta,membrane protein,285, t -> c, S, NA
orf8.fasta,membrane protein,292, g -> a, N, V -> I
orf8.fasta,membrane protein,297, t -> g, S, NA
orf8.fasta,membrane protein,396, a -> g, S, NA
orf8.fasta,membrane protein,414, t -> a, S, NA
orf8.fasta,membrane protein,435, c -> a, S, NA
orf8.fasta,membrane protein,438, t -> g, S, NA
orf8.fasta,membrane protein,440, c -> t, N, A -> V
orf8.fasta,membrane protein,456, c -> t, S, NA
orf8.fasta,membrane protein,459, c -> t, S, NA
orf8.fasta,membrane protein,462, c -> t, S, NA
orf8.fasta,membrane protein,468, c -> g, N, I -> M
orf8.fasta,membrane protein,513, t -> a, S, NA
orf8.fasta,membrane protein,516, t -> a, S, NA
orf8.fasta,membrane protein,525, c -> t, S, NA
orf8.fasta,membrane protein,538, a -> t, N, T -> S
orf8.fasta,membrane protein,540, a -> g, N, T -> S
orf8.fasta,membrane protein,547, t -> c, N, C -> L
orf8.fasta,membrane protein,548, g -> t, N, C -> L
orf8.fasta,membrane protein,552, a -> c, S, NA
orf8.fasta,membrane protein,575, g -> a, N, R -> K
orf8.fasta,membrane protein,630, t -> c, S, NA
orf8.fasta,membrane protein,634, t -> c, S, NA
orf8.fasta,membrane protein,639, t -> a, S, NA
orf8.fasta,membrane protein,648, t -> a, N, H -> Q
orf8.fasta,membrane protein,654, c -> t, S, NA
orf8.fasta,membrane protein,657, a -> t, S, NA
orf8.fasta,membrane protein,661, a -> t, N, M -> L
orf9.fasta,nucleocapsid protein,7, t -> w, N, F -> X
orf9.fasta,nucleocapsid protein,9, c -> t, N, F -> X
orf9.fasta,nucleocapsid protein,18, c -> t, S, NA
orf9.fasta,nucleocapsid protein,24, g -> a, S, NA
orf9.fasta,nucleocapsid protein,25, c -> t, N, P -> S
orf9.fasta,nucleocapsid protein,30, c -> t, S, NA
orf9.fasta,nucleocapsid protein,84, t -> c, S, NA
orf9.fasta,nucleocapsid protein,96, g -> a, S, NA
orf9.fasta,nucleocapsid protein,128, t -> c, N, V -> A
orf9.fasta,nucleocapsid protein,133, t -> c, N, S -> P
orf9.fasta,nucleocapsid protein,173, c -> a, N, T -> N
orf9.fasta,nucleocapsid protein,174, t -> c, N, T -> N
orf9.fasta,nucleocapsid protein,272, g -> k, N, G -> X
orf9.fasta,nucleocapsid protein,273, a -> w, N, G -> X
orf9.fasta,nucleocapsid protein,286, g -> r, N, E -> X

orf9.fasta,nucleocapsid protein,316, a -> c, S, NA
orf9.fasta,nucleocapsid protein,345, t -> c, S, NA
orf9.fasta,nucleocapsid protein,355, c -> t, S, NA
orf9.fasta,nucleocapsid protein,372, c -> t, S, NA
orf9.fasta,nucleocapsid protein,381, c -> t, S, NA
orf9.fasta,nucleocapsid protein,384, g -> t, S, NA
orf9.fasta,nucleocapsid protein,397, t -> c, N, Y -> H
orf9.fasta,nucleocapsid protein,411, t -> g, N, H -> Q
orf9.fasta,nucleocapsid protein,414, c -> t, S, NA
orf9.fasta,nucleocapsid protein,437, t -> a, N, F -> Y
orf9.fasta,nucleocapsid protein,465, t -> c, S, NA
orf9.fasta,nucleocapsid protein,484, g -> c, N, V -> L
orf9.fasta,nucleocapsid protein,486, c -> t, N, V -> L
orf9.fasta,nucleocapsid protein,495, t -> c, S, NA
orf9.fasta,nucleocapsid protein,552, a -> g, S, NA
orf9.fasta,nucleocapsid protein,584, t -> c, N, V -> A
orf9.fasta,nucleocapsid protein,601, a -> g, N, T -> A
orf9.fasta,nucleocapsid protein,617, a -> g, N, N -> S
orf9.fasta,nucleocapsid protein,625, c -> t, N, P -> S
orf9.fasta,nucleocapsid protein,631, t -> g, N, S -> A
orf9.fasta,nucleocapsid protein,642, t -> c, S, NA
orf9.fasta,nucleocapsid protein,670, t -> c, N, S -> P
orf9.fasta,nucleocapsid protein,693, c -> t, S, NA
orf9.fasta,nucleocapsid protein,721, c -> t, S, NA
orf9.fasta,nucleocapsid protein,753, t -> a, S, NA
orf9.fasta,nucleocapsid protein,783, g -> a, S, NA
orf9.fasta,nucleocapsid protein,784, g -> a, N, V -> I
orf9.fasta,nucleocapsid protein,792, a -> g, S, NA
orf9.fasta,nucleocapsid protein,831, c -> t, S, NA
orf9.fasta,nucleocapsid protein,921, c -> t, S, NA
orf9.fasta,nucleocapsid protein,1010, c -> t, N, S -> L
orf9.fasta,nucleocapsid protein,1068, g -> a, S, NA
orf9.fasta,nucleocapsid protein,1083, c -> t, S, NA
orf9.fasta,nucleocapsid protein,1089, c -> t, S, NA
orf9.fasta,nucleocapsid protein,1098, c -> t, S, NA
orf9.fasta,nucleocapsid protein,1101, a -> g, S, NA
orf9.fasta,nucleocapsid protein,1107, t -> a, S, NA
orf9.fasta,nucleocapsid protein,1116, g -> s, N, V -> X
orf9.fasta,nucleocapsid protein,1117, c -> t, S, NA
orf9.fasta,nucleocapsid protein,1119, t -> g, S, NA
orf9.fasta,nucleocapsid protein,1122, c -> t, S, NA
orf9.fasta,nucleocapsid protein,1123, c -> g, N, Q -> X
orf9.fasta,nucleocapsid protein,1125, g -> k, N, Q -> X
orf9.fasta,nucleocapsid protein,1137, c -> a, S, NA
orf9.fasta,nucleocapsid protein,1140, t -> c, S, NA
orf9.fasta,nucleocapsid protein,1146, t -> a, N, H -> Q
orf9.fasta,nucleocapsid protein,1158, g -> t, S, NA
orf9.fasta,nucleocapsid protein,1164, t -> g, N, I -> M
orf9.fasta,nucleocapsid protein,1170, t -> g, N, I -> M
orf9.fasta,nucleocapsid protein,1176, t -> a, S, NA

orf9.fasta,nucleocapsid protein,1206, t -> c, S, NA
orf9.fasta,nucleocapsid protein,1214, t -> g, N, V -> G
orf9.fasta,nucleocapsid protein,1228, g -> a, N, V -> I
orf9.fasta,nucleocapsid protein,1241, t -> c, N, V -> A
orf9.fasta,nucleocapsid protein,1245, t -> c, S, NA
orf9.fasta,nucleocapsid protein,1271, g -> t, N, S -> I
orf9.fasta,nucleocapsid protein,1275, a -> g, S, NA
orf9.fasta,nucleocapsid protein,1284, t -> a, S, NA
orf9.fasta,nucleocapsid protein,1325, a -> t, N, Y -> F
orf9.fasta,nucleocapsid protein,1335, t -> c, S, NA
orf10.fasta,N2 protein,23, t -> c, N, I -> T
orf10.fasta,N2 protein,35, g -> a, N, S -> N
orf10.fasta,N2 protein,67, t -> c, N, F -> L
orf10.fasta,N2 protein,72, t -> c, S, NA
orf10.fasta,N2 protein,112, c -> a, N, L -> T
orf10.fasta,N2 protein,113, t -> c, N, L -> T
orf10.fasta,N2 protein,211, g -> k, N, E -> X
orf10.fasta,N2 protein,212, a -> w, N, E -> X
orf10.fasta,N2 protein,225, g -> r, N, L -> X
orf10.fasta,N2 protein,255, a -> c, S, NA
orf10.fasta,N2 protein,284, t -> c, N, I -> T
orf10.fasta,N2 protein,294, c -> t, S, NA
orf10.fasta,N2 protein,311, c -> t, N, T -> I
orf10.fasta,N2 protein,320, c -> t, N, T -> I
orf10.fasta,N2 protein,323, g -> t, N, W -> L
orf10.fasta,N2 protein,336, t -> c, S, NA
orf10.fasta,N2 protein,350, t -> g, N, I -> S
orf10.fasta,N2 protein,353, c -> t, N, T -> M
orf10.fasta,N2 protein,376, t -> a, N, S -> T
orf10.fasta,N2 protein,404, t -> c, N, L -> S
orf10.fasta,N2 protein,423, g -> c, N, L -> F
orf10.fasta,N2 protein,425, c -> t, N, S -> L
orf10.fasta,N2 protein,434, t -> c, N, I -> T
orf10.fasta,N2 protein,491, a -> g, N, K -> R
orf10.fasta,N2 protein,523, t -> c, N, F -> L
orf10.fasta,N2 protein,540, a -> g, S, NA
orf10.fasta,N2 protein,556, a -> g, N, I -> V
orf10.fasta,N2 protein,564, c -> t, S, NA
orf10.fasta,N2 protein,570, t -> g, S, NA
orf10.fasta,N2 protein,581, t -> c, N, V -> A
orf10.fasta,N2 protein,609, t -> c, S, NA
